# A Randomized Trial Examining Housing First in Congregate and Scattered Site Formats

**DOI:** 10.1371/journal.pone.0168745

**Published:** 2017-01-11

**Authors:** Julian M. Somers, Akm Moniruzzaman, Michelle Patterson, Lauren Currie, Stefanie N. Rezansoff, Anita Palepu, Karen Fryer

**Affiliations:** 1 Somers Research Group Faculty of Health Sciences Simon Fraser University Burnaby, Canada; 2 School of Population and Public Health University of British Columbia Vancouver, Canada; Public Library of Science, FRANCE

## Abstract

**Objective:**

No previous experimental trials have investigated Housing First (HF) in both scattered site (SHF) and congregate (CHF) formats. We hypothesized that CHF and SHF would be associated with a greater percentage of time stably housed as well as superior health and psychosocial outcomes over 24 months compared to treatment as usual (TAU).

**Methods:**

Inclusion criteria were homelessness, mental illness, and high need for support. Participants were randomised to SHF, CHF, or TAU. SHF consisted of market rental apartments with support provided by Assertive Community Treatment (ACT). CHF consisted of a single building with supports equivalent to ACT. TAU included existing services and supports.

**Results:**

Of 800 people screened, 297 were randomly assigned to CHF (107), SHF (90), or TAU (100). The percentage of time in stable housing over 24 months was 26.3% in TAU (reference; 95% confidence interval (CI) = 20.5, 32.0), compared to 74.3% in CHF (95% CI = 69.3, 79.3, p<0.001) and 74.5% in SHF (95% CI = 69.2, 79.7, p<0.001). Secondary outcomes favoured CHF but not SHF compared to TAU.

**Conclusion:**

HF in scattered and congregate formats is capable of achieving housing stability among people experiencing major mental illness and chronic homelessness. Only CHF was associated with improvement on select secondary outcomes.

**Registration:**

Current Controlled Trials: ISRCTN57595077.

## Introduction

Housing First (HF) has been implemented internationally to promote recovery among people leaving homelessness with serious mental illness [1–4]. HF involves the provision of supports to clients in market housing (i.e., scattered among existing rental accommodations) with a strong emphasis on the promotion of client choice regarding the process of recovery, including sobriety and engagement with treatment [5; 6]. Outcomes of HF include robust positive impacts on residential stability [7], service costs [8;9], and client satisfaction [1]. The results of multi-centre randomised controlled trials have reported significant differences in housing stability between scattered site HF and usual care, but found an absence of differences on a wide range of secondary and exploratory outcomes including: quality of life; symptom severity; community integration (psychological and physical components); overall recovery; and community functioning [10;11].

As an alternative to the use of scattered sites, congregate HF (i.e., where all accommodations in a building are reserved for program clients) has been implemented in the US [12; 13], Europe [14], and Australia [15:^16^]. HF in congregate format has produced effective clinical outcomes and cost savings with clients with histories of homelessness and alcohol dependence [17; 18] and has been hypothesized to offer advantages to participants with complex needs including substance dependence [19]. In some jurisdictions, the co-location of clients in a single site may be seen as preferable based on potential efficiencies and economies of scale. However, little is known about the impact of congregate HF on overall recovery or the relative benefits of congregate HF and scattered site HF for clients with mental illness and co-occurring substance use disorders. No experimental trials have investigated these questions. The current study addresses this gap by examining data from a randomised controlled trial which compared the effectiveness of scattered site HF (SHF) and congregate HF (CHF) versus treatment as usual (TAU) for adults with histories of chronic homelessness, current mental illness, and high levels of need for support in Vancouver BC.

The Vancouver At Home study is part of a five-site Canadian project investigating scattered site interventions for people who are both homeless and mentally ill. The five sites shared a common core of measures, and the related outcomes have been reported (10,11). In addition, each site expanded on the common core in order to address distinct research questions related to homelessness and mental illness. In Vancouver, a unique focus was the inclusion of HF in both congregate and scattered site formats.

Aims of the Study: We hypothesized that both SHF and CHF would generate superior outcomes than TAU over 24 months on housing stability (primary outcome) and on the following secondary outcomes: community functioning; community integration; quality of life; recovery; food security; and psychiatric symptom severity. Participants met criteria for longstanding homelessness, serious mental illness, and a high level of need for support.

## Methods

### Study design and participants

This study was a non-blinded, parallel three-arm randomised trial [20]. Recruitment was conducted with community-based partners (n = 40) representing homeless shelters, outreach teams, mental health and addiction service providers, hospitals, police and justice system diversion programs. Research ethics board approval was received from Simon Fraser University and the University of British Columbia.

Verbal consent was obtained to conduct eligibility screening. Interviews were conducted by trained researchers. Eligible individuals were: at least 19 years old; met criteria for at least one current mental disorder; were absolutely homeless or precariously housed; had moderate or severe disability defined as a score of 62 or lower on the Multnomah Community Ability Scale (MCAS;[21]), as well as at least one of the following: legal system involvement in the past year, substance dependence in the past month, or two or more hospitalizations for mental illness in any one of the past five years. Homelessness was defined as either absolute homelessness (having no place to sleep or live for more than 7 nights and little likelihood of obtaining accommodation in the coming month) or precarious housing (currently residing in marginal accommodation and having two or more episodes of absolute homelessness as defined above in the past year). Current mental illness was assessed using the Mini International Neuropsychiatric Interview 6.0 (MINI; [22]) for the following: major depressive episode; manic or hypomanic episode; post-traumatic stress disorder; mood disorder with psychotic features; and psychotic disorder. Interviewers assessed participants’ mental status (e.g., current substance use or psychiatric symptoms) and rescheduled interviews if indicated. Written informed consent was obtained from all participants, with recruitment extending from October 2009 to June 2011.

### Randomisation

Randomisation was performed using a centralized computer generated procedure. Interviewers used laptop computers with secure live connections to upload data and receive randomisation results prior to notifying participants of the outcome. Randomisation results were received by interviewers after baseline interviews were completed, and participants randomised to SHF or CHF were directed immediately to service representatives.

### Procedures

Services were modeled on the approach developed by Pathways to Housing (PH), including an emphasis on promoting client choice and adoption of a harm reduction ethos and practices in relation to addiction [6]. Training was delivered to service providers by senior personnel from PH. Two structured fidelity assessments were conducted by an external team [6], comprised of representatives from PH, the study funder, and individuals who had experienced homelessness.

For SHF, an inventory of private market rental apartments was developed in a variety of neighborhoods throughout the city of Vancouver. A maximum of 20% of the units in any building could be allocated to the study and participants were provided with a choice of housing units [6]. A housing portfolio manager was responsible for building and maintaining relationships with landlords. Participants in the SHF condition received support in their homes from an Assertive Community Treatment (ACT) team. The CHF condition had on site 24x7 supports comparable to ACT and was mounted in a single vacant building with the capacity to house at least 100 occupants in independent suites but without full kitchens. The building was located in a mixed residential and commercial neighborhood, adjacent to numerous amenities, and was equipped with facilities to support residents, including: central kitchen and meal area, medical examination room and formulary, and recreational areas (yoga, basketball, road hockey, lounge). Tenants were provided with opportunities to engage in part-time work both within the building (e.g., meal preparation, laundry) and in the community (e.g., graffiti removal service). A reception area and front desk were staffed 24 hours. Tenancy in either of the experimental housing conditions was not contingent on compliance with specific therapeutic objectives (e.g., addiction treatment). Program staff in each intervention condition participated in a series of continuing professional development events in person. Subsidies were provided through the study to ensure that participants paid no more than 30% of their total income on rent. Treatment as Usual (TAU) consisted of existing services and supports available to homeless adults with mental illness living in Vancouver.

A team of field interviewers followed participants. Interviewers received in-depth training and supervision in the administration of measures, which were pre-tested with a sample of participants. Interviews were considered ‘on time’ if they occurred within 2 weeks of the designated due date. Participants received C$35 for the baseline interview and C$20–30 for each subsequent interview. Scales were administered in person at 6-month intervals through 24 months and responses entered immediately on laptop computers. Additional brief interviews every 3-months collected details of residential and vocational time-lines. Interviews conducted at 6-month intervals required between 90 to 180 minutes to complete in most cases. A field research office was open daily throughout the study period, and participants were encouraged to drop-in regardless of their interview schedule. Interviewers obtained periodic updates regarding participants’ routines and typical whereabouts, and collateral contact information was obtained in order to facilitate future follow up. Interviews were conducted in various locations based on randomisation arm and participant preference, including participants’ homes, the field research office, and public settings.

### Outcomes

The primary outcome for the trial was housing stability over 24 months, based on the percentage of time stably housed, obtained using the Residential Time-Line Follow-Back Inventory (RTLFB). The RTLFB has demonstrated strong psychometric properties in homeless samples [23]. We administered the scale every 3-months in order to enhance accuracy of recall, and participants’ residence status and type was coded for each day during the recall period. As a result we generated a continuous record of housing status for each participant throughout the trial. We defined stable housing on the basis of holding a lease (i.e., tenancy rights) or living in one’s own residence (room, apartment, house or with family) for an expected duration of at least six months. Participants who were living in other housing conditions (the streets, emergency shelters, crisis units, hospitals, jails, etc.) were considered as unstably housed.

Secondary outcomes and their associated instruments were: severity of disability (Multnomah Community Ability Scale (MCAS) [21]), community integration (Community Integration Scale (CIS) [24]); psychiatric symptom severity (Colorado Symptom Index (modified) (CSI) [25]); overall health (EuroQol 5D (EQ-5D) [26]); food security (USDA Adult Food Security Survey Module [27]); substance use (Global Appraisal of Individual Needs, Substance Problem Scale (GAIN-SPS) [28]); quality of life (Quality of Life Interview, 20-item (QoLI-20) [29]); and recovery (Recovery Assessment Scale, 22-item (RAS-22) [30]). Scales for secondary outcomes were administered at 6-month intervals [20], however comparisons were made based on difference scores between Baseline and study end. Safety and adverse events were monitored throughout the study.

### Statistical analysis

The primary outcome analysis involved separate comparisons of SHF and CHF with TAU on an end point analysis of housing stability. Our sample size estimate was based on a moderate effect size for the primary outcome (Cohen’s d = 0.5) with significance levels of 0.05 (two-tailed). With no attrition rate and no adjustment for multiplicity, a sample of 64 participants in each study arm would have sufficient power (80%) (Ref 20; 2). The formula (n_new_ = n/1-L) is used to estimate the adjusted sample (n_new_) to account for the attrition rate (L). With a multiplicity adjustment (two pairwise comparisons: CHF vs. TAU & SHF vs. TAU) and an attrition rate of 10%, the estimated sample size was 87 in each arm. A recruitment target of 100 participants in each arm was planned anticipating a higher attrition rate.

All analyses were based on an intention-to-treat principle. The primary outcome (percentage of time stably housed) was calculated using total number of days in stable residences over 24 months following randomisation as numerator and total number of days in any type of residence (stable or unstable) during the same time period as denominator. Secondary outcome analyses compared change scores, which were calculated as the difference between 24-month and baseline assessments on each measure. Due to the continuous nature of outcome variables, one-way analysis of variance (ANOVA) was used. Following ANOVA, post-hoc pairwise comparisons (SHF vs. TAU and CHF vs. TAU) were performed to evaluate the intervention effect. Dunnett’s method was used to correct for multiple comparisons resulting from the multi-arm study design with a single comparison group [31]. If Levene’s test for homogeneity of variance was non-significant (p ≥ 0.05), the overall p value was based on ANOVA test and adjusted p values for pairwise comparisons (SHF vs. TAU and CHF vs. TAU) were based on Dunnett’s test. If Levene’s test was significant (p < 0.05), the overall p value was based on Welch’s ANOVA test and adjusted p values for pairwise comparisons were based on Games-Howell test. As measures of the intervention effect, difference scores (percentage of time stably housed over 24-month for the primary outcome, and change scores between baseline and 24 months relevant scales for secondary outcomes) between specific HF and TAU along with 95% confidence intervals were reported. All reported p values were two sided. Because groups were balanced in terms of baseline characteristics [20], outcome analysis with adjustment of covariates was not performed.

Missing data were observed in this study due to invalid (e.g., ‘declined,’ ‘do not know’) or skipped responses to specific items/scales, and participants who died, withdrew or were lost to follow up. Missing data for the primary outcome was low (2%) and higher for secondary outcomes (see S1 Table: Follow up completion rate for secondary outcomes). Last observation was carried forward.

For certain instruments (Food Security, GAIN-SPS, RAS), the response ‘Do not know’ was considered negative or neutral, as appropriate, and used as a valid response to calculate the total scale score. To replace missing responses to specific items, mean substitution was used to obtain the total scores as long as no more than half of the items were missing. Missing baseline values were replaced by the group specific mean.

Sensitivity analysis was conducted among participants with non-missing outcome data and the same analytic method. IBM SPSS Statistics (Version 22.0) was used to conduct these analyses.

This trial is registered with Current Controlled Trials: ISRCTN57595077 (Vancouver at Home Study: Housing First plus Assertive Community Treatment versus Congregate Housing plus Supports versus treatment as usual). In order to protect participant anonymity, the data used in the following analyses are not publically available. Data access requests can be made by contacting Karen Fryer at kfryer@sfu.ca.

## Results

**Fig 1:**
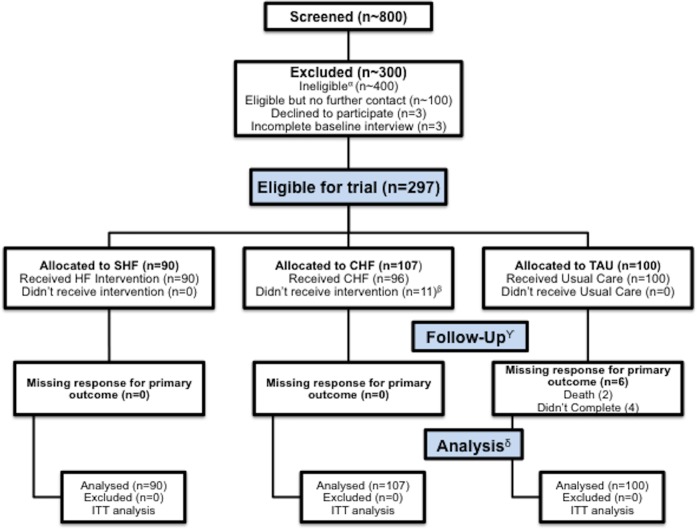
Participant flow through screening, assessment, allocation to study arm, completion of follow-up visits and inclusion in the analysis.

A participant flow diagram is shown in the Figure (Fig 1, Participant Flow). A total of 800 individuals were screened and 297 met eligibility criteria and were randomised. In most cases, exclusion was due to ineligibility. The first participant was enrolled on October 19, 2009 and the final participant was enrolled on June 29, 2011. The follow up rate (291 out of 297 participants) for the primary outcome variable (percentage of time stably housed) at 24 months was 98% (SHF: 100%, CHF: 100%, TAU: 94%). Missing data were due to participant deaths (n = 3, within five months of randomisation) and failure to locate participants (n = 3). The number of participant deaths over 24 months (n = 17) did not differ significantly between groups (SHF: 7; CHF: 4; TAU: 6; Log-rank p value = 0.482, see S2 Table: Mortality among ‘Vancouver At Home’ Participants (n = 297) by study arms), but missing data due to follow-up were higher in the TAU arm.

Participant baseline characteristics by study arm are shown in Table 1. Participants were roughly 40 years old, predominantly male and White, and had not completed high school. Psychiatric status reflected study inclusion criteria, with the majority meeting criteria for a Psychotic Disorder as well as Substance Dependence. On average, participants first experienced homelessness in their mid-twenties, had been homeless for at least 3 years cumulatively, and most reported three or more comorbid physical illnesses.

**Table 1:** Characteristics of VAH participants at enrolment visit (randomization).

Variable	CHF (n = 107) N %	SHF (n = 90) N (%)	TAU (n = 100) N (%)
**Socio-Demographics**			
Age at randomization (years), mean (SD)	40.0 (11.6)	39.5 (10.8)	39.5 (11.2)
Male gender	82 (77)	66 (74)	70 (71)
Ethnicity			
• Aboriginal	21 (20)	11 (12)	12)
• White	60 (56)	53 (59)	57)
• Mixed/Other	26 (24)	26 (29)	31 (31)
Incomplete high school	70 (66)	47 (53)	62 (62)
Single/Never married	76 (72)	63 (70)	75 (77)
**Homelessness, median (IQR)**			
Lifetime duration of homelessness (months)	36 (12–72)	42 (12–84)	48 (13–109)
Longest duration of homelessness (months)	20 (7–48)	12 (6–40)	12 (6–48)
Age of first homelessness (years)	27 (20–39)	26 (19–35)	24 (18–36)
Absolutely homeless, n (%)	88 (82)	72 (80)	72 (72)
**MINI International Neuropsychiatric Interview diagnosis**			
Major Depressive Episode	35 (33)	31 (34)	29 (29)
Manic or Hypomanic Episode	25 (23)	23 (26)	20 (20)
Post-Traumatic Stress Disorder	27 (25)	17 (19)	19 (19)
Panic Disorder	20 (19)	15 (17)	24 (24)
Mood Disorder with psychotic feature	20 (19)	17 (19)	19 (19)
Psychotic Disorder	79 (74)	59 (66)	73 (73)
Alcohol dependence	28 (26)	19 (21)	25 (25)
Substance dependence	67 (63)	55 (61)	61 (61)
Suicidality (moderate or high)	34 (32)	28 (31)	31 (31)
Daily drug use	31 (29)	19 (21)	32 (32)
Injection drug use	19 (18)	16 (18)	19 (20)
**Comorbid Conditions List (CMC)**^**1**^			
Blood-borne infectious diseases^2^	33 (32)	23 (26)	31 (32)
Head Injury	66 (62)	62 (69)	63 (63)
Multiple (≥ 3) physical illness	69 (65)	52 (58)	68 (68)
**Secondary/exploratory outcome**^**3**^^**,**^^**4**^			
Severity of disability (MCAS)	49.90 (6.69)	51.64 (6.52)	50.63 (6.98)
Physical community integration (CIS)	2.10 (1.75)	1.64 (1.47)	1.83 (1.70)
Psychological community integration (CIS)	10.61 (3.68)	11.29 (3.48)	11.10 (3.19)
Psychiatric symptom severity (CSI)	37.12 (12.91)	36.40 (13.34)	40.25 (12.49)
Overall health (EQ5D)	59.48 (23.58)	64.22 (22.65)	62.04 (22.07)
Food security (FS)	4.24 (2.54)	4.29 (2.56)	4.79 (2.41)
Substance use problems (GAIN-SPS)	2.39 (1.94)	2.09 (1.88)	2.29 (1.92)
Quality of life (QOLI20)	72.61 (21.69)	76.22 (21.20)	74.72 (21.43)
Recovery (RAS-22)	76.83 (11.26)	80.18 (11.14)	78.85 (10.53)

CI: Confidence Interval; CIS: Community Integration Scale; CHF: Congregate Housing First; EQ5D: EuroQuol 5D; GAIN-SPS: Global Assessment of Individual need–Substance Problem Scale; ITT: Intention-To-Treat; INT: Intervention; MCAS: Multnomah Community Ability Scale; QOLI20: Quality of Life Index 20 Item; RAS-22: Recovery Assessment Scale 22 item; SHF: Scattered Site Housing First; TAU: Treatment As Usual; VAH: Vancouver At Home.

1. Response ‘Do not know’ was considered as no.

2. Includes HIV, Hepatitis C & Hepatitis B.

3. Missing values were replaced by group mean.

4. Levene’s test for homogeneity of variance was non-significant and p value was obtained from One-way ANOVA with equal variance.

### Primary outcome

During the 24-month follow-up period, the percentage of time spent in stable housing was significantly higher in both intervention arms compared to TAU (see Table 2). Using the intent to treat sample (n = 297), the intervention effect (mean difference between intervention and TAU condition) was 48.0% (95% Confidence Interval (CI) = 40.0–56.3) for CHF and 48.2% (95%CI = 39.5–56.9) for SHF. Intervention effects using the non-missing sample (n = 291) were 46.4% (95%CI = 37.9–54.8) for CHF and 46.5% (95%CI = 37.7–55.3) for SHF.

**Table 2:** Effect of Housing First Intervention on Primary Outcome (percentage of days in stable housing) among VAH participants (n = 297).

	Number of days in stable residence Mean (SD)	Total number of days with housing data^1^ Mean (SD)	% of time spent in stable residences Mean (95% CI)	P value for overall comparisons^2^	Intervention effect: difference in % of stable housing (Intervention -TAU) Mean (95% CI)	Adjusted P value^3^ for pairwise comparisons
**ITT sample (n = 297)**^**4**^						
CHF (n = 107)	509.3 (195.0)	676.1 (116.8)	74.3 (69.3, 79.3)	**<0.001**	48.0 (40.0, 56.3)	**<0.001**
SHF (n = 90)	509.0 (188.3)	684.1 (109.2)	74.5 (69.2, 79.7)		48.2 (39.5, 56.9)	**<0.001**
TAU (n = 100)	181.1 (204.5)	650.6 (164.2)	26.3 (20.5, 32.0)		Reference	Reference
**Non-missing sample (n = 291)**						
CHF (n = 107)	509.3 (195.0)	676.1 (116.8)	74.3 (69.3, 79.3)	**<0.001**	46.4 (37.9, 54.8)	**<0.001**
SHF (n = 90)	509.0 (188.3)	684.1 (109.2)	74.5 (69.2, 79.7)		46.5 (37.7, 55.3)	**<0.001**
TAU (n = 94)	192.7 (205.6)	650.6 (169.4)	27.9 (22.0, 33.9)		Reference	Reference

CI: Confidence Interval; CHF: Congregate Housing First; ITT: Intention-To-Treat; SHF: Scattered Site Housing First; TAU: Treatment As Usual; VAH: Vancouver At Home.

1. Total number of days with housing data didn’t differ significantly between groups.

2. Levene’s test for homogeneity of variance was non-significant and p value was obtained from One-way ANOVA with equal variance.

3. Dunnet’s test was used to adjust for family-wise errors.

4. -Six participants had missing information and were treated as being still homeless for ITT analysis.

### Secondary outcomes

Treatment effects on secondary outcomes are presented in Table 3.

**Table 3:** Effect of Housing First Intervention on Secondary/exploratory Outcomes among VAH participants (n = 297).

							Intervention effect				
ITT sample	24-month			Change of score (24-month–baseline)			Difference in change of score (INT-TAU)		P value^2^^,^^3^		
(n = 297)^1^	Mean (SD)			Mean (95% CI)			Mean (95% CI)				
	CHF	SHF	TAU	CHF	SHF	TAU	CHF	SHF	Overall	CHF	SHF
Severity of disability (MCAS)^4^	68.08 (9.18)	65.69 (9.36)	63.01 (9.81)	18.19 (16.27, 20.11)	14.04 (11.81, 16.28)	12.38 (10.48, 14.29)	5.81 (2.69, 8.93)	1.66 (-1.59, 4.92)	**<0.001**	**<0.001**	0.418
Physical community integration (CIS)	2.82 (1.90)	1.36 (1.49)	2.07 (1.79)	0.72 (0.32, 1.11)	-0.28 (-0.67, 0.10)	0.24 (-0.15, 0.64)	0.47 (-0.14, 1.09)	-0.53 (-1.16, 0.11)	**0.002**	0.152	0.122
Psychological community integration (CIS)	14.66 (3.70)	12.46 (3.58)	12.62 (3.70)	4.05 (3.15, 4.94)	1.18 (0.08, 2.27)	1.52 (0.63, 2.40)	2.53 (1.05, 4.01)	-0.34 (-1.88, 1.20)	**<0.001**	**<0.001**	0.840
Psychiatric symptom severity (CSI)	26.25 (10.98)	27.67 (10.81)	27.70 (11.80)	-10.87 (-13.42, -8.32)	-8.73 (-11.37, -6.09)	-12.55 (-15.31, -9.78)	1.68 (-2.44, 5.80)	3.82 (-0.49, 8.12)	0.145	0.567	0.090
Overall health (EQ5D)	68.57 (20.22)	68.63 (19.97)	69.80 (18.58)	9.09 (3.62, 14.56)	4.42 (-0.66, 9.50)	7.76 (2.81, 12.71)	1.33 (-6.74, 9.40)	-3.34 (-11.78, 5.09)	0.444	0.907	0.583
Food security (FS)	3.58 (2.11)	4.40 (2.50)	3.91 (2.18)	-0.66 (-1.27, -0.04)	0.11 (-0.52, 0.74)	-0.88 (-1.52, -0.25)	0.23 (-0.74, 1.20)	0.99 (-0.02, 2.01)	0.079	0.822	0.057
Substance use problems (GAIN-SPS)	1.34 (1.67)	1.18 (1.72)	1.00 (1.57)	-1.05 (-1.51, -0.59)	-0.91 (-1.36, -0.46)	-1.29 (-1.71, -0.88)	0.24 (-0.44, 0.93)	0.38 (-0.34, 1.10)	0.486	0.647	0.392
Quality of life (QOLI20)	91.80 (24.55)	93.82 (23.77)	87.80 (22.71)	19.19 (14.34, 24.05)	17.60 (11.95, 23.25)	13.09 (8.01, 18.16)	6.11 (-1.91, 14.12)	4.51 (-3.86, 12.89)	0.220	0.161	0.382
Recovery (RAS-22)	86.31 (15.29)	84.13 (11.10)	82.75 (10.79)	9.47 (6.81, 12.14)	3.95 (1.53, 6.37)	3.90 (1.96, 5.83)	5.58 (1.65, 9.50)	0.05 (-3.63, 3.74)	**0.0025**	**0.008**	0.999

CI: Confidence Interval; CIS: Community Integration Scale; CHF: Congregate Housing First; EQ5D: EuroQuol 5D; GAIN-SPS: Global Assessment of Individual need–Substance Problem Scale; ITT: Intention-To-Treat; INT: Intervention; MCAS: Multnomah Community Ability Scale; QOLI20: Quality of Life Index 20 Item; RAS-22: Recovery Assessment Scale 22 item (RAS-22); SHF: Scattered Site Housing First; TAU: Treatment As Usual; VAH: Vancouver At Home.

1. We used the Last Observation Carried Forward (LOCF) to replace the missing 24-month values.

2.If Levene’s test for homogeneity of variance was non-significant (p <0.05), the overall p value was based on ANOVA test and adjusted p values for pairwise comparisons (CHF vs. TAU and SHF vs. TAU) were based on Dunnet’s test.

3. Levene’s test for homogeneity of variance was non-significant for all outcome variables except RAS-22 score.

4. Higher score superior for MCAS, CIS, EQ5D, QOLI20, RAS-22. Lower score superior for CSI, FS, GAIN-SPS.

5. Since Levene’s test for homogeneity of variance for RAS-22 core was significant, the overall p value was based on Welch ANOVA test and p values for pairwise comparisons were based on Games-Howell test.

The mean change in MCAS score (severity of disability) from baseline to 24 months was significantly different between TAU and CHF participants (5.81, 95%CI = 2.69–8.93), but not between TAU and SHF participants (1.66, 95%CI = -1.59–4.92).

Mean change from baseline to 24 months did not differ significantly between SHF and TAU for community integration on physical (0.47, 95%CI = -0.14–1.09) or psychological subscales (-0.34, 95%CI = -1.88–1.20), psychiatric symptom severity (3.82, 95%CI = -0.49–8.12), overall health (-3.34, 95%CI -11.78–5.09), substance use problems (0.38, 95%CI = -0.34–1.10), community functioning (1.66, 95%CI = -1.59–4.92), quality of life (4.51, 95%CI = -3.86–12.89), or recovery (0.05, 95%CI = 3.63–3.74). A difference approaching significance (p = 0.057) was observed for food security and favouring TAU compared to SHF at 24 months (0.99, 95%CI = -0.02–2.01).

Mean change from baseline to 24 months was significantly greater in CHF compared to TAU for psychological community integration (2.53, 95%CI = 1.05–4.01) and recovery (5.58, 95%CI = 1.65–9.50). No differences between CHF and TAU were observed for physical community integration (0.47, 95%CI = -0.14–1.09), psychiatric symptoms (1.68, 95%CI = -2.44–5.80), overall health (1.33, 95%CI = -6.74–9.40), food security (0.99, 95%CI = 0.02–2.01), substance problems (0.24, 95%CI = -0.44–0.93), or quality of life (6.11 (95%CI = -1.91–14.12). The same significant differences favouring CHF were obtained with analyses restricted to non-missing cases (see S3 Table: Sensitivity analysis (non-missing cases) for effect of Housing First Intervention on Secondary Outcomes among VAH participants).

## Discussion

HF in both congregate (CHF) and scattered site (SHF) formats achieved markedly superior housing stability compared with TAU over the 24-month follow-up period. Previous studies have reported high rates of housing stability through SHF for people with mental illnesses [32] and CHF for people with alcohol dependence [17]. The current study is the first experimental trial to compare SHF alongside CHF with usual care. Our results demonstrate the nearly equivalent housing stability outcomes associated with both interventions for homeless adults with serious mental illness and comorbid conditions including substance dependence.

We found no evidence of improvement relative to TAU in SHF on any of the secondary outcomes examined. These null findings are consistent with the results of a recent multi-site randomised trial of SHF involving participants selected on the basis of less severe needs [10] as well as an earlier multi-site study reporting that chronically homeless and mentally ill individuals were successfully rehoused yet remained socially isolated with limited improvement in social integration [33]. In contrast, the current trial found that CHF was associated with significant improvement concerning severity of disability, psychological community integration, and recovery. The measures detecting these differences respectively assess subjective experiences of community belonging and participation [23;34;35], subjective appraisal of psychiatric and physical health [36–38], and interviewer assessed level of functioning across multiple domains [21;39]. These secondary outcomes may be interpreted as hypothesis generating and await further research and replication.

Although both SHF and CHF had equivalent complements of service providers, the team supporting SHF provided outreach throughout the city on (at least) a weekly basis. The team supporting CHF worked on site and was able to engage residents as indicated. Additional factors that may have contributed to improvement in CHF were on-site recreational and vocational opportunities, and a supportive peer environment. Qualitative research has found that ongoing substance use and experiences of loneliness and isolation are often reported following the transition to SHF [40–42]. Difficulties transitioning to SHF may explain some of the null findings compared with TAU over 24 months. In contrast, previous research on CHF has identified that shared backgrounds and experiences of residents contributed to a positive sense of community [18].

Analyses of administrative data in the current trial have shown that participants randomised to SHF and CHF interventions had fewer criminal convictions [43] and fewer emergency department visits [44] than those assigned to TAU. Qualitative analyses identified substantial deficiencies in shelter and support services in TAU [42]. Notwithstanding these previous findings, the current results indicate that neither CHF or SHF were sufficient to mediate changes over 24 months in measures of quality of life, overall health, or psychiatric symptom severity, beyond what would be expected from prolonged homelessness with minimal supports. Attention is needed on adaptations to HF that stimulate change in these domains, and on identifying and acting on the factors that predict youth at risk for prolonged homelessness [45–48;49].

At baseline our sample had high prevalence of psychosis (71%) and substance dependence (62%)[20], which are associated with very high mortality risk among the homeless [50;51]. Seventeen participants died during the 24 month follow up, whereas several previous trials of SHF reported no participant deaths over at least 24 months [5;22;52]. We observed no differences in rates of death between study arms, demonstrating that intensive inter-disciplinary interventions were not sufficient to significantly reduce the likelihood of mortality compared to usual care.

Limitations of this research include reliance on self-report. Notwithstanding this limitation, comparison of self-report and administrative data sources within our sample (for justice, health, and social services) revealed high overall levels of agreement [53]. A further limitation is that we are unable to account for potential neighbourhood-level effects in our analyses (i.e., while SHF apartments were dispersed throughout Vancouver, the CHF intervention was necessarily in a single neighbourhood). Our sample of mentally ill homeless people may not be representative of populations served in other locations. Secondary outcome analyses should be considered exploratory and hypothesis generating. Strengths include an experimental design, well-funded HF with independent fidelity assessments, 24-month follow up, and strong participant retention [20].

Previous research suggests that individuals with active psychosis may respond less favourably to CHF [18]. Further investigation is needed to examine whether individual level characteristics are associated with differing outcomes between CHF and SHF. HF is clearly capable of achieving high levels of housing stability. Nevertheless, recent trials have found that SHF has not resulted in client improvements across a wide range of additional outcomes over 24 months [10;11;54]. Research must now examine adaptations to HF that promote recovery following the advent of housing. The current study contributes to this goal by investigating the relative impact of SHF and CHF compared with TAU for people with serious mental illness, prevalent substance use, and multiple comorbidities.

## Supporting Information

S1 CONSORT Checklist (DOC)

S1 Table Follow up completion rate for secondary outcomes (6-month interval scale) (DOCX)

S2 Table Mortality among ‘Vancouver At Home’ Participants (n = 297) by Study Arms (DOCX)

S3 Table Sensitivity analysis (non-missing cases) for effect of Housing First Intervention on Secondary Outcomes among VAH participants (DOCX)

S1 Protocol (PDF)
