# Supplementary material for: A Randomized Trial Examining Housing First in Congregate and Scattered Site Formats
Source: PLoS One. 2017 Jan 11;12(1):e0168745. doi: 10.1371/journal.pone.0168745 (PMC5226665; doi:10.1371/journal.pone.0168745)
Supplement: S1 Table — (DOCX) [file pone.0168745.s002.docx]

S1 Table: Follow up completion rate for secondary outcomes (6-month interval scale)

|  | Overall | CHF | SHF | TAU |
| --- | --- | --- | --- | --- |
| 6-month visit, n (%) | 281 (95) | 103 (96) | 88 (98) | 90 (90) |
| 12-month visit, n (%) | 274 (93) | 103 (96) | 85 (94) | 86 (86) |
| 18-month visit, n (%) | 257 (87) | 96 (90) | 84 (93) | 77 (77) |
| 24-month visit, n (%) | 250 (84) | 97 (91) | 81 (90) | 72 (72) |
| At least one follow-up (6-month interval) visit | 287 (97 | 105 (98) | 89 (99) | 93 (93) |
| Reason for no follow up visit (n=10 | 5 (1.6) | 1 (1) | 1 (1) | 3 (3) |
| Death1 | 5 (1.6) | 1 (1) | 0 (0) | 4 (4 |
| No contact |  |  |  |  |

CHF: Congregate Housing First; SHF: Scattered Site Housing First; TAU: Treatment as Usual

1. Total number of deaths (n=17) was as follows: CHF-4, SHF-7 & TAU-6. However, 12 participants completed at least one follow up visit before death and the remaining 5 participants whose follow up data was not available died within seven months of randomization.
