# Supplementary material for: A Randomized Trial Examining Housing First in Congregate and Scattered Site Formats
Source: PLoS One. 2017 Jan 11;12(1):e0168745. doi: 10.1371/journal.pone.0168745 (PMC5226665; doi:10.1371/journal.pone.0168745)
Supplement: S2 Table — (DOCX) [file pone.0168745.s003.docx]

S2 Table: Mortality among ‘Vancouver At Home’ Participants (n=297) by Study Arms

|  | **Death1** | **Total Person-Years (PYs)2** | **Death per 100 PYs** | **p value3** |
| --- | --- | --- | --- | --- |
| **Study Arms** |  |  |  |  |
| **CHF** | 4 | 287.3 | 1.4 | 0.482 |
| **SHF** | 7 | 226.5 | 3.1 |  |
| **TAU** | 6 | 262.7 | 2.3 |  |
| **Total** | 17 | 776.5 | 2.2 |  |

1. Among 17 cases, cause of deaths was known for 7 participants (Overdose of drug or alcohol –2,

Cancer-1, Suicide -1, Heart diseases- 1, Respiratory diseases- 1 & HIV- 1).

2.-Patients who died were censored at the time of death and all other participants who didn’t die were censored at study end (March 31, 2013).

3. Log-rank was used to compare survival curves between study arms.
