# Supplementary material for: A Randomized Trial Examining Housing First in Congregate and Scattered Site Formats
Source: PLoS One. 2017 Jan 11;12(1):e0168745. doi: 10.1371/journal.pone.0168745 (PMC5226665; doi:10.1371/journal.pone.0168745)
Supplement: S3 Table — (DOCX) [file pone.0168745.s004.docx]

S3 Table: Sensitivity analysis (non-missing cases) for effect of Housing First Intervention on Secondary Outcomes among VAH participants

|  |  | | |  | | | **Intervention effect** | | | | |
| --- | --- | --- | --- | --- | --- | --- | --- | --- | --- | --- | --- |
| **Non-missing case1** | **24-month Mean (SD)** | | | **Change of score (24-month–baseline)\ Mean (95% CI)** | | | **Difference in change of score (INT-TAU) Mean (95% CI)** | | **P value2** | | |
|  | CHF | SHF | TAU | CHF | SHF | TAU | CHF | SHF | Overall | CHF | SHF |
| Physical community integration (CIS) (n=249) | 2.86 (1.92) | 1.36 (1.52) | 2.08 (1.73) | 0.68 (0.25, 1.10) | -0.30 (-0.73, 0.12) | 0.15 (-0.32, 0.62) | 0.53 (-0.17, 1.23) | -0.45 (-1.18, 0.28) | **0.006** | 0.163 | 0.282 |
| Psychological community integration (CIS) (n=249) | 14.72 (3.77) | 12.51 (3.56) | 12.82 (3.60) | 4.03 (3.08, 4.98) | 1.34 (0.20, 2.48) | 1.59 (0.60, 2.59) | 2.44 (0.81, 4.07) | -0.25 (-1.96, 1.45) | **<0.001** | **0.002** | 0.920 |
| Psychiatric symptom severity (CSI) (n=240) | 25.20 (10.36) | 27.79 (11.38) | 26.89 (11.72) | -11.97 (-14.58, -9.36) | -9.15 (-11.97, -6.33) | -14.60 (-17.84, -11.36) | 2.63 (-1.90, 7.16) | 5.45 (0.74, 10.15) | **0.038** | 0.327 | **0.020** |
| Overall health (EQ5D) (n=244) | 69.18 (20.96) | 70.67 (17.46) | 70.38 (17.21) | 8.92 (2.72, 15.12) | 5.93 (0.71, 11.15) | 9.43 (3.23, 15.59) | 0.51 (-9.89, 8.87) | -3.50 (-13.23, 6.24) | 0.679 | 0.989 | 0.633 |
| Food security (FS) (n=245) | 3.54 (2.13) | 4.50 (2.53) | 4.04 (2.33) | -0.73 (-1.38, -0.08) | 0.24 (-0.44, 0.93) | -0.99 (-1.79, -0.19) | 0.26 (-0.85, 1.38) | 1.23 (0.07, 2.40) | **0.043** | 0.816 | **0.036** |
| Substance use problems (GAIN-SPS) (n=246) | 1.32 (1.66) | 1.11 (1.69) | 0.90 (1.43) | -1.15 (-1.65, -0.65) | -0.95 (-1.41, -0.49) | -1.56 (-2.03, -1.09) | 0.41 (-0.36, 1.19) | 0.61 (-0.19, 1.42) | 0.234 | 0.382 | 0.162 |
| Severity of disability (MCAS) (n=249) | 68.62 (8.73) | 66.38 (8.82) | 64.75 (9.29) | 18.64 (16.72, 20.56) | 14.85 (12.60, 17.10) | 14.18 (12.05, 16.31) | 4.46 (1.15, 7.77) | 0.67 (-2.79, 4.13) | **0.005** | **0.006** | 0.871 |
| Quality of life (QOLI20) (n=243) | 92.47 (24.38) | 94.82 (23.72) | 87.35 (21.35) | 19.48 (14.38, 24.58) | 18.74 (12.60, 24.89) | 15.62 (9.81, 21.45) | 3.85 (-5.11, 12.81) | 3.12 (-6.18, 12.41) | 0.613 | 0.526 | 0.670 |
| Recovery (RAS-22) (n=221) | 90.09 (14.75) | 84.54 (10.48) | 84.37 (9.88) | 12.83 (9.51, 16.15) | 4.68 (1.83, 7.53) | 5.91 (3.08, 8.73) | 6.95 (2.08, 11.78) | -1.23 (-6.12, 3.67) | **<0.001** | **0.003** | 0.796 |

CI: Confidence Interval; CIS: Community Integration Scale; CHF: Congregate Housing First; EQ5D: EuroQuol 5D; GAIN-SPS: Global Assessment of Individual need –Substance Problem Scale; ITT: Intention-To-Treat; INT: Intervention; MCAS: Multnomah Community Ability Scale; QOLI20: Quality of Life Index 20 Item; RAS-22: Recovery Assessment Scale 22 item (RAS-22); SHF: Scattered Site Housing First; TAU: Treatment As Usual; VAH: Vancouver At Home

1. Non-missing cases varies across scale (221 to 249)

2. Levene’s test for homogeneity of variance was non-significant for all outcome variables (p <0.05). The overall p value was based on ANOVA test and adjusted p values for pairwise comparisons (CHF vs. TAU and SHF vs. TAU) were based on Dunnet’s test.
