# Supplementary material for: A Randomized Trial Examining Housing First in Congregate and Scattered Site Formats
Source: PLoS One. 2017 Jan 11;12(1):e0168745. doi: 10.1371/journal.pone.0168745 (PMC5226665; doi:10.1371/journal.pone.0168745)
Supplement: S1 Protocol — (PDF) [file pone.0168745.s005.pdf]

Research Demonstration Project on Homelessness & Mental Health:

Research Design for Two Randomized Controlled Trials in Vancouver, British Columbia

Table of Contents

Table of Contents ii
1.0 Principal Investigators 1
2.0 Engagement of People with Lived Experience 1
3.0 Innovation and Relevance of the Study 2
3.1 Rationale 2
3.2 Background 3
4.0 Research Design 5
4.1 Hypotheses 6
4.2 Eligibility Criteria 7
4.3 Recruitment 7
4.4 Interventions 8
4.4.1 Housing First 8
4.4.2 Assertive Community Treatment (ACT) 10
4.4.3 Intensive Case Management (ICM) 11
4.4.4 Congregate Housing with Supports: 12
4.4.5 Treatment as Usual (TAU) 14
4.5 Follow-up and Retention Procedures 14
4.6 Outcome Measures and Timelines 14
4.6.1 Physical and Mental Health 15
4.6.2 Psychosocial Determinants and Consequences of Change 15
4.6.3 Cost Outcome Analyses & Administrative Data 17
4.6.4 Additional Outcomes of Interest 18
4.7 Good Clinical Practice 18
4.8 Data Integrity and Management 18
4.9 General Approach to Data Analysis 18
5.0 Ethical Considerations 21
5.1 Quantitative Data 21
5.2 Qualitative Data. 22
5.3 Administrative Data. 23
5.4 Procedures for REB approval 23
6.0 Sustainability 23
7.0 Integrated Knowledge Translation Strategy 24
7.1 Media and Communications 25
8.0 Project Structure and Governance 25
8.1 Committees/Teams 25
8.2 Scientific Advisors 26
8.3 Project Management Capacity/Structure 26
8.4 Project Organization Chart 26
8.5 Roles and Responsibilities of Stakeholders and Project Team 27
9.0 Workplan 28
10.0 Budget (See Attached Spreadsheets) 30
11.0 References (See Appendix 6.0) 30

1.0 Principal Investigators

- Julian M Somers, PhD, R.Psych. (Lead Applicant) Associate Professor, Simon Fraser University
- Michael Krausz, MD, PhD, FRCPC, Professor, University of British Columbia
- James Frankish, PhD, Professor, University of British Columbia

Three Principal Investigators provide research leadership and interdisciplinary expertise to the Vancouver consortium. The Lead Applicant (JS) is Director of the Centre for Applied Research in Mental Health and Addiction (CARMHA) and Associate Professor in the Faculty of Health Sciences at SFU. Both co-PIs are Professors at UBC, with appointments at the School of Population and Public Health (JF) and in Psychiatry and at the Centre for Health Evaluation and Outcome Studies (MK). All PIs have extensive experience directing research in mental health, homelessness, and addiction, including leading randomized controlled trials (MK), directing multi-site longitudinal interventions (JS and MK), and maintaining programs of qualitative and mixed-methodological research (JS, JF, MK).

Current or recent relevant initiatives involving the PIs include the NAOMI RCT; the German Heroin Trial; estimating the housing needs of British Columbians with severe mental disorders; and evaluating the impacts of service innovations designed to improve outcomes for homeless people with mental disorders (e.g., drug treatment court; residential treatment for concurrent disorders; intensive supervision of criminal offenders with severe mental disorders). The PI's are actively engaged as advisors to branches of government and agencies dedicated to addressing the needs of homeless individuals with mental disorders, including the Victoria Mayor's Task Force (JS, MK), the City of Vancouver's Collaboration for Change (JS, MK), the Streetohome Foundation (JF, JS), the BC Ministries of Health and Housing & Social Development (JS) and Vancouver Coastal Health Authority (JF, MK, JS).

SFU will serve as the administrative home for the proposed research. CARMHA's offices are located in downtown Vancouver, and will provide the physical operating environment for project implementation, including established facilities and procedures for the secure stewardship of sensitive data.

Consortium members include additional academics from major universities, provincial and municipal decision-makers, key decision-makers from the Vancouver Coastal Health, Providence Health Care, the City of Vancouver, and key community housing and mental health service providers. All agencies and members have significant experience working on collaborative research and/or service delivery projects at the provincial and/or national level. All members are committed to the project principles and objectives and have been actively involved in the application development process. [Please see Addendum for CVs of Principal Investigator, co-PIs, Co-Investigators and Service Leads.] [Please see Appendix 1.0 for complete list of Consortium members and profiles of Service Lead organizations.]

2.0 Engagement of People with Lived Experience

The engagement of people with lived experience (PWLE) is critical to the planning and development of this project. Six round table sessions were held in December 2008/January 2009 to begin the process of inviting PWLE to participate in the project. These conversations informed the development of the application and how to meaningfully engage PWLE throughout the project. In total, 58 PWLE commented on the project design and shared their perspectives on issues for further research consideration as well as ways they could continue to contribute to the study (see Summary in Appendix 2.0). An engagement strategy for PWLE has been developed that outlines both direct and supportive roles, as well as processes and partnerships that create space for PWLE to remain at the centre of the

project in a number of Advisory, Research, Public Stakeholder, Service Provision and Project Infrastructure capacities. (See Engagement Strategy in Appendix 2.1)

3.0 Innovation and Relevance of the Study

3.1 Rationale

Over the past 30 years, there has been significant downsizing of long-stay psychiatric institutions, accompanied by inadequate investments in the growth and integration of community services responsible for mental disorders, substance use disorders, welfare, justice and housing. As a result, many cities, including Vancouver, have witnessed a significant increase in the number of homeless individuals with mental disorders (including addictions) with diverse housing and health-related needs that are complicated by physical health problems, trauma, and various social and occupational challenges.

Untreated psychiatric and physical health conditions both contribute to chronic homelessness and to poor quality of life. However, the services designed to address mental health, addiction and physical health are often segregated and incomplete. Physical health conditions tend to be treated in Emergency Departments where continuity of follow-up care is limited. On the other hand, mental health and addictions are most often addressed by diverse community-based agencies (Folsom et al., 2005); however, service agencies are often ill-equipped to address the multiple needs of individuals with concurrent mental and substance use disorders, possibly leading to incomplete care and further unmet need. Given the high rates of physical and behavioural health problems among homeless individuals and the inadequacy of services, there is a growing need for effective approaches that integrate housing with treatment and support services (Rosenheck et al., 2003).

A growing body of research demonstrates that supported housing has a positive impact on residential stability, regardless of the specific model of housing (Best, 2006; Rog, 2004). Recent work has indicated that a Housing First approach, which provides permanent, independent housing that is scattered throughout the community, is an effective approach for people who are homeless with mental disorders, including substance use (e.g., Tsemberis & Eisenberg, 2000). This model places few treatment demands on clients and provides intensive support services to help them integrate into their community. Despite this work, the impacts of supported housing on outcomes other than those related to residential stability and hospitalization have not been consistently studied, and existing studies have not yielded consistent results. A Cochrane review of supported housing for people with severe mental disorders (Chilvers, Macdonald & Hayes, 2008) found no studies that met the selection criteria (randomization). Furthermore, few, if any, studies of supported housing have incorporated randomization to ‘usual care’ that did not include some form of housing. Addressing this gap in the research literature is critical for the development of policies and well-defined intervention programs.

Given the shortage of affordable housing units, especially in urban areas, the implementation of scattered-site housing may not lead to the most effective use of available housing resources. Policymakers therefore should explore the relative advantages and disadvantages of various alternative strategies, including approaches that encourage re-housing of homeless persons with severe mental illness in *congregate* settings that include other persons. Few studies to date have examined the effect of moving into congregate arrangements on persons with severe mental disorders (see O’Flaherty et al., in progress; Walker & Seasons, 2002).

Evidence is emerging to suggest the characteristics of effective interventions for homeless individuals with psychiatric symptoms, including the importance of perceived choice (Greenwood et al., 2005; Nelson et al., 2007). *Assertive Community Treatment (ACT)* is a model of care for people with severe mental illness in which a multidisciplinary team provides treatment and rehabilitation in addition to case management functions. An extensive body of research has shown that ACT is effective in reducing hospitalization and improving symptoms of mental illness as well as social functioning (see Ziguras & Stuart, 2000). *Intensive Case Management (ICM)* is another model of care for people with mental illness in which services are brokered to community agencies by a case manager rather than delivered by a team (as in ACT). The evidence base for ICM is not as strong as that for ACT; however, it has been shown to be effective in improving symptoms of mental illness as well as social functioning (see Dixon & Goldman, 2003). Focusing on a subset of homeless people with severe mental illness, Coldwell and Bender (2007) used meta-analysis to assess the effectiveness of ACT versus standard case management. Overall, clients who received ACT had a 37% reduction in homelessness and a 26% improvement in psychiatric symptom severity compared to standard case management. These results suggest that homeless people with highly disabling conditions may best be served by a service model such as ACT.

Despite the body of evidence in favour of ACT and, to a lesser degree, ICM, little is known regarding the effectiveness of different intensities of intervention for homeless individuals with differing levels of need. For example, are the ACT and ICM models effective for individuals with concurrent disorders? Can ACT-like services be effectively integrated into a congregate housing setting? What models of care are effective for homeless people with moderate needs? It is our hope that the evidence gathered through the proposed trials will promote an informed evolution away from reactive programs (e.g., shelters, emergency medicine) and toward long-term, community-based solutions.

3.2 Background

In Vancouver, the overlap between mental disorders, substance use, and homelessness has become a civic crisis. When compared to the rest of British Columbia and Canada, Vancouver is unique in terms of the heterogeneity, multi-morbidity and concentration of its homeless population. The extent of chronic medical conditions, including infectious disease, has historically been well-documented among Vancouver's homeless population (Acorn, 1993; Wood, Kerr et al., 2003). Furthermore, many homeless individuals are not connected to the formal health care system, and are at elevated risk of adverse outcomes, including overdose (Kerr et al., 2005).

The 2008 Metro Vancouver Homeless Count found 1,372 people who were homeless in the City of Vancouver[1]. This number of homeless represents a 23% increase since the previous count in 2005. Notably, between 2005 and 2008, the percentage of people who have experienced homelessness for a year or more increased by 65%, representing 48% of people counted in 2008. In addition, over this period, self-reports of both mental illness and addictions increased significantly, by 86% and 63% respectively. A 2007 Provincial estimate of the population of adults with severe mental disorders (including substance use disorders) estimated that 1,800 adults in Vancouver are absolutely homeless and an additional 2,280 adults are at-risk for homelessness (see Figure 1 in Appendix 3.0; Somers, 2008). These reports suggest not only a significant increase in the rates and severity of homelessness in Vancouver, but that a substantial number of people are affected.

[1] The 2008 Metro Vancouver Homeless Count also identified an additional 1,037 homeless individuals in suburban areas adjacent to the City of Vancouver.

Vancouver's Downtown East Side (DTES) is home to about 16,000 residents. Vancouver Coastal Health (n.d.a) estimated that 3,200 individuals in the DTES have significant health problems and an additional 2,100 have more substantive disturbances that require intensive support and services. However, other estimates suggest an even greater level of need: Eby and Misura (2006) estimated that 5,000 injection drug users are infected with Hepatitus C or HIV/AIDS. Many infected individuals do not receive treatment for their conditions other than medical care through Emergency Departments (Kerr et al, 2005). Furthermore, many of these individuals are homeless or live in unstable housing conditions.

The high concentration of Single Room Occupancy (SRO) hotels in the DTES is also unique to the Vancouver context and has contributed to the urban ghettoization of poverty, mental illness and addiction. A high demand for low income housing is evidenced by the 0.5% vacancy rate for bachelor suites in Vancouver. As a result, affordable housing is far beyond the shelter allowance of people receiving income assistance. The average rent for a bachelor apartment is $736/month, almost double the $375 monthly shelter allowance. In response to the growing levels of homelessness and the related issues in health and social problems, several non-profit organizations have established housing and other supportive services, many of which are in the DTES.

Estimates of the clinical, social and housing service needs within the population of people who are homeless with mental disorders vary widely. Evidence from other urban centres suggests that about one-third of homeless persons have a mental disorder (e.g., Toro, 2007). Goering et al. (2002) found that 64% of first-time shelter users in Toronto had a history of drug abuse and 64% had other psychiatric problems. For those who had previously used shelters, 71% had drug abuse histories and 69% had other psychiatric problems. Despite a lack of precision and consistency among these results, it is clear that mental and substance use disorders are highly prevalent among the homeless. The variability and complexity of need within the homeless population requires evidence-based interventions that respond to individuals with both high and moderate levels of need. Furthermore, diverse behavioural and public health services (e.g., needle exchange, consumption facilities) are in need of expansion. Although Provincial ACT Standards have been developed and a Provincial Advisory Committee has been established to initiate ACT province-wide, currently, there are no Assertive Community Treatment (ACT) teams in Vancouver, and only two in all of BC.

In general, housing in Vancouver for people with multiple barriers due to substance use and other mental disorders has been in *congregate* settings, and this trend is continuing with the purchase and renovation of a number of SROs and the development of congregate housing on 12 city sites. Growing civic commitment and public concern in Vancouver has been directed toward improving the health, autonomy, and quality of life among those who are homeless and have mental disorders. Nevertheless, progress in providing services for this population is hampered by limited resources. Agencies and institutions have also struggled to overcome differences of organizational cultures, mandates and styles of work. Indeed, the preparation of this application has encouraged diverse stakeholder groups to work together and establish a common framework. For the RDP to be successful and, ultimately, for the country to gain the knowledge needed to provide effective services to people who are homeless, a philosophy of shared leadership among high-performance teams that transcend organizational boundaries is vital.

Vancouver's newly elected Mayor has struck a Task Force to address the issue of homelessness. Numerous city- and province-led initiatives have recently addressed challenges related to homelessness, including reforms to the justice system (e.g., Community Court), expanded mental health services (e.g., Burnaby Centre for Mental Health & Addiction), access to income assistance (e.g.,

Homeless Outreach Teams), and investments to stabilize housing stock (e.g., purchase of SROs and development of additional supportive housing). If these activities and commitments fulfill their promise, they will significantly improve the standard of "usual care" for homeless people with mental disorders in Vancouver.

In summary, this proposal addresses a critical gap in the research evidence surrounding housing and services for a growing population of vulnerable individuals. In light of the limited housing resources and options available in Vancouver, this study not only responds to the MHCC's national multi-site initiative, but offers a unique opportunity to evaluate an alternative model of services that may be more feasible and sustainable. Alternative models are important to consider in light of concerns about a growing economic crisis.

4.0 Research Design

The proposed research design includes the utilization of randomized controlled trial methods in order to evaluate integrated health and housing services. The project involves two sub-populations of homeless individuals, characterized by different eligibility criteria: one group with "high needs" and a second group with "moderate needs". The project thus consists of two related but distinct RCTs which differ with regard to the severity of participants' needs, the number of intervention arms, and the type of intervention services. Despite these differences, many of the major methodological features of each RCT will be the same. These include: participant recruitment and retention strategies, tests and measures of outcomes of interest, study hypotheses, and plans for data analysis. In addition, both RCTs incorporate qualitative and quantitative methods along with the secondary analysis of administrative data.

Participants with high needs will be randomized to one of two *Housing First* program variations (Scattered-site + ACT or Congregate + ACT*) or to a Treatment as Usual (TAU) group (see Figure 2). Participant choice will be maximized in all study conditions, and all study participants who are randomized to an intervention group will receive some form of low-barrier housing as well as intensive support services. Moderate need participants will be randomized to one of two groups, an intervention (Scattered-site + ICM) or TAU group. Please see Section 4.4 for details on the housing and support interventions as well as the TAU group.

Figure 2. Study Group Interventions by Need

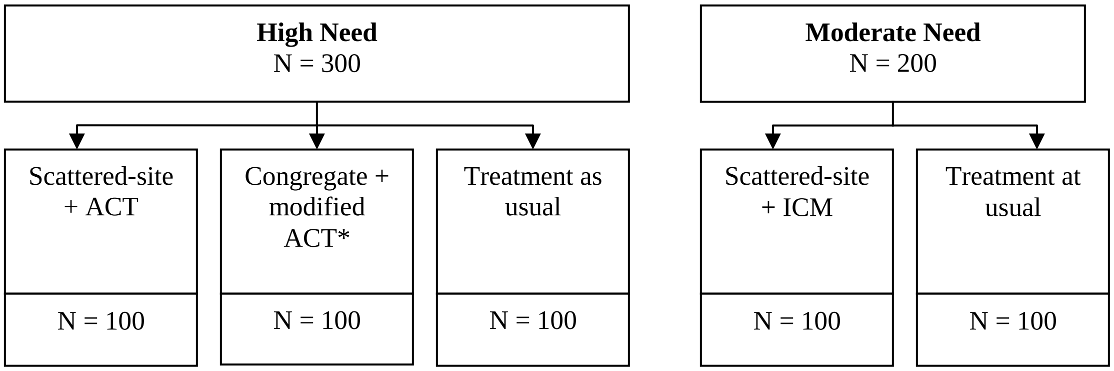

*Intensive treatment services will be integrated into the congregate housing condition. In the event that an ACT team cannot be developed in this condition, the service component will consist of a comparable range and intensity of services.

All study participants will be followed and re-interviewed every 6 months for a period of 2 years from baseline. A field office will be established to facilitate tracking and interviewing of participants. The field office will be utilized as a confidential location for conducting follow-up interviews as well as a place where participants can ask questions about the project, their next interview (e.g., date, time, etc.), and update their contact information.

Overall, the design follows accepted standards, including adherence to Good Clinical Practice (GCP). In conformity to GCP, it is necessary to develop Standard Operating Procedures, an audit protocol, and trials monitoring procedures. These details are not presented in the present application, pending the further specification of treatment components (e.g., ICM) and resolution of additional design details across sites. The unique elements of the Vancouver study site will also be articulated and submitted for trials registration to complement the activities registered as part of the multi-site trial.

4.1 Hypotheses

This study provides a rich opportunity for examining a broad range of hypotheses. The key primary and secondary hypotheses are outlined below.

Primary hypotheses:

In comparison to individuals in the Treatment as Usual groups, individuals assigned to Housing First groups will:

(1) attain ***stable housing***; and
(2) realize improved ***mental health*** outcomes in terms of both number and severity of symptoms.

Secondary hypotheses:

In comparison to individuals in the Treatment as Usual groups, and when compared to their own baseline (within subjects), individuals assigned to Housing First groups will demonstrate:

(1) improved general ***quality of life***;
(2) a different distribution of service ***costs*** (and the component services themselves) between the health, justice, and income assistance systems;
(3) attenuation of ***trauma symptoms***;
(4) reduced ***victimization*** (financial, sexual, physical);
(5) reduced incidents of ***harm*** to self and harm to others;
(6) reduced rates of ***justice*** system involvement (i.e., police contacts, Provincial court sentences);
(7) increased ***employment*** and more stable receipt of appropriate income assistance benefits; and
(8) improved ***physical health.***

In comparison to individuals in the Treatment as Usual group and, when compared to their own baseline (within subjects), individuals assigned to Housing First + ACT groups (incorporating contingency management) will demonstrate:

(9) reduced ***substance use*** and related problems.

In addition to the above, a final hypothesis is that:

(10) local ***media*** accounts of homelessness and mental illness will change over the course of the project in frequency and quality (e.g.: reduced stigma; more promising regarding potential solutions), based on analyses of newsprint and video.

4.2 Eligibility Criteria

Criteria for inclusion in the project are as follows: (1) Legal adult status (age 19 in BC); (2) Meets definition of 'absolute homelessness' or 'precariously housed' set by MHCC; (3) Presence of any serious mental disorder with or without a concurrent substance use disorder. (N.B. Formal diagnosis is not required at time of entry into the project.)

Participants will be excluded from the study if they do not meet any of the inclusion criteria and if any of the following conditions are met: (1) Inability to communicate in English; (2) Incarceration or institutionalization (current or imminent); (3) Incompetent to give informed consent; or (4) Refusal to comply with the research protocol.

4.3 Recruitment

Recruitment will rely on referral from a variety of sources (e.g., Community Court; mental health and addiction services; Emergency Departments; shelters) and outreach. Outreach workers and service providers will be asked to inform homeless people about the project. Recruitment materials (e.g., posters, brochures) will be distributed in the community and displayed in organizations that serve the target population (e.g., soup kitchens, shelters, drop-in centres, health clinics, etc.). Recruitment will also rely on word-of-mouth among friends and associates ("snowballing"). The research team will conduct information sessions with community organizations and professionals who serve the target population to inform them about the project and gain support. Specific organizations that target women and ethnic minorities will be contacted to encourage diversity in the sample. Screening will be conducted via laptop computer within community agencies and the field office will be used for baseline and follow-up interviewing.

Recruitment to the full sample is estimated to take 18 months (1 year following a 6 month implementation phase). However, two potential issues related to our ability to recruit participants may extend this period: (1) the randomization process, and (2) compliance with the research protocol. Participants may perceive randomization to intervention and comparison groups as being 'good' or 'bad,' respectively, possibly leading to refusal to participate. Further, some participants may be hesitant to comply with the rigors of the protocol.

Screening and Baseline Evaluation: The screening process will consist of two primary steps:

1. **Pre-Screening**: Due to the nature of chronic homelessness in the DTES, it is likely that most individuals will have an addiction and/or mental illness (Goldner et al., 2007). Thus, the basic criteria needed to conduct pre-screening will be age 19 years or older, competence to sign the screening consent form, proficiency in spoken English, and *absolute homelessness* or *precarious housing* (as defined by the MHCC).
2. **Full Screening**: If pre-screening criteria are satisfied, participants will be asked the full set of screening questions. This will include evaluation of full inclusion and exclusion criteria, including classification as high or moderate need. Classification as *high*-need will correspond to Section 3 of Ontario Standards for ACT, as set forth by the MHCC. Classification as *moderate*-need will also be based on the same criteria; however, participants will be excluded if their score on the Symptom Checklist-90-R exceeds a threshold (to be determined) appropriate for the ICM condition. The

proportion of potential participants who fall into the high vs. moderate needs groups will be tested empirically during the implementation phase of the project[2].

Screening will be computer-based and will inform the interviewer whether or not the participant meets the study inclusion criteria as well as their level of need. Participants will be asked to return to the field office the following day to complete the **baseline evaluation** and then to receive notification of group assignment. In order to facilitate follow-up, participants will be asked to provide locations/ services they frequent regularly. This requirement to return the following day may pose a barrier to recruitment and recruiters will be trained to actively seek people who do not return.

Randomization will be performed separately for the two need groups (high and moderate). To control for initial differences among groups, randomization strata will include with or without substance use problems and absolute homelessness or precariously housed. Randomization will be performed using a block technique with variable block size using prepared tables. When participants return the next day to complete the baseline evaluation, they will receive notification of group assignment in a sealed envelope along with an information package; at this time, a field office worker will accompany participants who are assigned to an active intervention to the service provider's location. Those randomized to TAU will be informed about available services in the community. All participants will be invited to use the field office as a resource centre.

Blinding: It is not possible to blind the interventions given that participants, researchers and service providers will know the kind of intervention received. However, data analysts will be blind; a code will be provided for each condition that will not be identified until the end of the study.

4.4 Interventions

Given the study design, it will be important that each intervention maintains fidelity to its key ingredients. The Housing First Fidelity Scale (Tsemberis et al., in preparation) will be used with all intervention sites to assess fidelity to the principles of Housing First. The Dartmouth ACT Scale (DACTS; Teague, Bond & Drake, 1998) will be used to assess the fidelity of the ACT team to the principles of this program. Any modifications made for the target population will also be included in the fidelity assessment (see Tsemberis, 1999; Drake, McHugo, et al., 1998). Fidelity criteria for ICM and Congregate interventions will be developed in concert with other study sites. Given that none of the proposed service models operate in isolation, it will be important to assess the sufficiency of resources in the community. Finally, for all clinical interventions, Standard Operating Procedures will be established according to the guidelines of GCP to ensure standardized provision of care.

4.4.1 Housing First

Housing First is an well-documented approach that focuses on housing people directly from the street in scattered-site, independent units; provides services that are client-centred and solution-focused; and provides a range of innovative programs designed to meet the needs specific to homeless people (Tsemberis & Eisenberg, 2000). Scattered-site units (200+) in Vancouver will be developed by the Motivation, Power & Achievement (MPA) Society across the city through partnerships with private landlords, and local permanent housing agencies including BC Housing. For over 15 years, MPA has

[2] There are some concerns that the OSACT criteria may be inadequate given the severity of mental illness and substance use in Vancouver's homeless population, possibly resulting in under-sampling of moderate-need individuals.

been establishing relationships with a large and varied portfolio of landlords and currently administers 188 private market rental housing subsidies.

Mechanisms for Administering Housing Subsidies: Rent will be paid directly to landlords on behalf of clients on a specified date each month. The client's portion of rent will be sent directly to MPA from the BC Ministry of Human Resources and/or calculated based on income. MPA will also administer damage deposits on behalf of clients, and facilitate the return of deposits and the cost of repairs to landlords, if necessary. A program-specific rental database will be created and maintained. MPA will ensure flexibility of rent supplements should clients move addresses; provide key contacts in accounts payable to respond to landlord queries; and provide monthly reporting of client rent subsidies. Based on the experience of Pathways to Housing, Inc. (a well-established non-profit organization that provides Housing First to people who are homeless in New York City), we anticipate that 15-20% of people housed in scatter-site housing will require at least one relocation over the course of the project.

Plan to Secure a Housing Portfolio: A challenge for the Vancouver site will be in securing sufficient scattered-site units during the recruitment phase (Sept. 2009-Aug. 2010) which coincides with the 2010 Winter Olympics. Strategies to address this challenge will be developed by MPA in collaboration with the Project Team, BC Housing, the City of Vancouver and other formal and informal partners. The following actions will be taken to secure an adequate housing portfolio for the project:

- Hire a full-time property and portfolio Manager under the direction of the Program Director;
- Work with BC Housing, the City of Vancouver and other non-profit social housing providers to secure housing units;
- Focus on portfolio development and relationship-building with landlords; build on existing relationships and support landlords towards successful tenancies; facilitate landlord round-tables and education forums, and form associations with landlord groups;
- Develop a dispute resolution process for landlords, and respond quickly to incidents via investigation, mediation and resolution;
- Engage people with lived experience in landlord education and relations;
- Develop procedures and checklists to ensure housing quality; monitor landlords' care and attention to clients; respect for tenancy agreements; etc.
- Support both ACT and ICM providers with immediate access to housing for clients; maintain ongoing communication with case workers and appropriate intervention with landlords;
- Liaise with Ministry of Human Resources regarding damage deposits and rent as necessary;
- Develop memos of understanding regarding roles and responsibilities with ACT and ICM leads.

With regard to access to housing facilities for the congregate intervention, discussions with BC Housing and the City of Vancouver regarding the provision or acquisition of sufficient congregate units have been ongoing. Both agencies are interested in continuing the dialogue and exploring partnerships around the availability of housing units within their respective portfolios. It is unclear whether congregate housing will consist of one building with 100 units or two buildings with a total of 100 units (where a significant number of units will be available by September 2009 and an additional number of units at a later time consistent with the overall period of recruitment). The Streetohome Foundation is also interested in exploring partnerships to ensure that a congregate intervention is possible. (See Appendix 4.0 for letters of support).

4.4.2 Assertive Community Treatment (ACT)

ACT will be delivered by Raincity Housing & Support Society (see Appendix 1.0 for profile).
Intake, Admission and Discharge: Raincity has significant experience in the assessment of clients' suitability and services needed for housing stability and independence. In partnership with the client, the ACT team will develop a detailed housing and service plan, based on a recovery orientation that respects the client's personal preferences and autonomy. Clients must agree to two requirements: (1) Rent paid directly to the landlord; (2) A minimum of one home visit a week for the duration of the project. Transition and discharge from housing and services will be coordinated among the ACT team and other agencies. It is anticipated that discharge from ACT will occur only in exceptional circumstances. The local ACT team will develop a process for discharge decisions similar to that developed by Pathways to Housing, Inc. which includes the following steps: (1) ACT team first makes a recommendation to discharge; (2) team and clinical director meet to discuss and explore options; (3) team meets with the client to discuss and explore options; (4) If discharge is confirmed then discharge proceeds with referral.

Service Intensity & Capacity: ACT teams use a multidisciplinary approach to provide intensive clinical services and supports directly to the client. The team will be closely involved in confirming housing and supporting all aspects of moving in and connecting to the local community. The client/staff ratio will be 10:1 or less and the team will be available 24 hours a day, 7 days a week, and 365 days per year to respond to client emergencies. At least 80% of services provided by the ACT team will be provided in the community. Client-centred assessment and individual treatment planning is a key ingredient of ACT. Clients' responses to interventions and supports are closely monitored so that interventions can be adjusted to meet changing needs. Close monitoring may include medication management; representative payeeships; and urine drug screens (Drake et al., 1993). The team will also be involved in any hospital admissions and discharges. We are currently developing a memo of understanding between the ACT team and St Paul's Hospital.

Staffing: The 8 to 12 member ACT team will consist of a social worker(s), substance abuse counselor, nurse(s), psychiatrist, peer specialist, family specialist, and employment specialist. The team will meet daily to review clients' status and jointly plan team members' daily activities. Team members will be cross-trained in each other's areas of expertise to the maximum extent feasible. Recruitment, hiring, training and staff orientation will occur during the study's implementation phase.

Program Organization & Communication: Services and team structure will adhere to the BC ACT Standards (MOHS, 2008) and guidelines provided by Pathways to Housing, Inc. The standard ACT model will be augmented in order to meet the needs of homeless individuals with concurrent mental and substance use disorders. For example, staff will be trained in contingency management, motivational enhancement and other strategies for managing poly-substance use. During the implementation phase, specific program procedures, communication materials and policies will be developed. Follow-up support will require multi-agency collaborations. Raincity has a long history of collaborative partnerships (e.g., addictions, mental health, primary care, other non-profits, police, Government and the local Health Authority) and will continue to build these relationships through presentations with community providers, the development of information and referral packages, and the coordination of regular meetings with outreach programs throughout the city. The ACT team will have access to information technology, meeting space, administrative services, fundraising, communications and human resources through Raincity.

Services Offered: ACT team services include: psychiatric treatment; substance abuse treatment; illness management; support with daily living skills including budgeting and money management skills; advocacy for benefits and entitlements; and community integration supports including linkages to health, wellness and recreational activities; family repatriation; vocational and supported employment services and supports. The team will also provide Contingency Management programs to address stimulant drug use. Other important approaches include enhancing motivation (e.g., motivational interviewing), teaching of cognitive-behavioral skills for relapse prevention, harm reduction and psychoeducation about addiction and concurrent disorders.

4.4.3 Intensive Case Management (ICM)

In this project, ICM will be delivered by Coast Mental Health, which has provided supported housing services to vulnerable populations since 1974. Through the formal implementation of outreach services as well as front-line advocacy, psychosocial and volunteer programs, they have a proven track-record in providing quality, client-centred services for people who are homeless and have mental health or substance use problems. (See Appendix 1.0 for profile).

ICM will be based on Toronto's Streets To Homes model, which provides intensive outreach and follow-up of homeless people. In ICM, services are brokered to other agencies by a Case Manager. Outreach workers work with clients to develop housing plans and facilitate housing continuity. Follow-up supports assist with community integration, life skills, and financial stability.

Intake, Assessment & Discharge: Clients with moderate needs who are randomized to this condition will be directed to the ICM team after they have completed the research screening and assessment. Coast has significant experience in the assessment of clients' suitability and the supports needed for housing stability and independence. In partnership with the client, the ICM team will develop a detailed housing and service plan that respects the client's personal preferences and autonomy. Clients must agree to two requirements: (1) Rent is paid directly to the landlord; (2) Once housed, regular contact with ICM staff for a minimum of one year. Transition and discharge from housing, if necessary, will be coordinated among the ICM team and other community and clinical agencies.

Service Intensity & Capacity: The client-to-staff ratio will be approximately 20:1 (individual caseloads), seven days per week, and 12 hours per day. In the event that intensive 1:1 support is necessary, it will be tapered as needed. If clients are struggling to maintain their housing, provisions and contingencies will be addressed by the team. The assessment will inform the level of support necessary. In general, in Phase I (months 0-4), the client and Case Manager meet at least twice per week. The primary goal of this stage is to help the client become familiar with their new community via community mapping. In Phase II (months 4-8), the frequency of meetings is reduced, if appropriate. In Phase III (months 9+), next steps will be discussed and planned with the client. Throughout this process, the Case Manager works closely with the landlord and other community service providers to ensure that the client is well supported.

Staffing: The ICM team will be comprised of experienced Coast personnel who transfer into the new positions as well as new hires with the required competencies. In additions to training provided by the National Project Team, Coast provides in-house training in concurrent disorders, non-violent crisis intervention, first aid and psychosocial rehabilitation skills. Coast also has a long history of including

and engaging people with lived experience in their program planning, as peer support workers and in other forms of paid employment.

Program Organization & Communication: Recruitment, hiring, training and staff orientation will occur during the project implementation phase. Specific program procedures, communication materials and policies will be developed and refined during this implementation phase. Follow-up support will require multi-agency collaborations. Coast has a long history of collaborative partnerships and will continue to build these relationships through presentations with community providers, the development of information and referral packages, and the coordination of regular meetings with outreach programs throughout the city. Coast is a large organization which has a comprehensive infrastructure to support a range of programs and services. The ICM team will have access to IT, meeting space, administrative services, fundraising, communications and HR

Services: Programming is based on a psychosocial rehabilitation/recovery model as well as strong harm reduction principles. The ICM model will broker services outside the team and will closely follow services provided through Coast's existing outreach teams, with further development in collaboration with other study sites (e.g., use of common tools and outcome measures such as the Outcome Star). Coast has established many services which clients from the ICM group could access, including meal and basic needs programs, employment and vocational programs, educational funds, training and employment in Coast Landscapes (social enterprise), access to recovery groups, art programs, volunteer opportunities, and various social programs.

The interventions for clients with concurrent disorders will include motivational enhancement strategies such as enhancing motivation for treatment and discussing the interactive effects of mental and substance use disorders; referral to providers of integrated substance abuse and mental health services or, if integrated services are not available or accessible, facilitating communication between separate brokered mental health and substance abuse service providers; and coordinating with community-based services to support the client's involvement in mutual self-help groups and outpatient treatment activities.

4.4.4 Congregate Housing with Supports:

Congregate housing and supports will be provided by the Portland Hotel Society (PHS). PHS has been operating since 1993 to serve individuals who are the hardest-to-house (e.g., those with mental illness, HIV, criminal history, substance abuse problems). PHS runs a number of innovative services including congregate, low-barrier housing. (See Appendix 1.0 for profile). The Congregate Housing with Supports condition was included given the difficulty of obtaining affordable rental stock in the current rental market (vacancy rate <1.0%) and in recognition that, in Vancouver, this is the dominant housing model for people who are homeless and mentally ill. Congregate housing will operate based on the Housing First model and will focus on building community acceptance.

Admission/discharge: Individuals who meet the project inclusion criteria for a high-level of need and are randomized assigned to Congregate Housing with Supports will be directed to PHS for intake. As in all conditions, tenant needs will drive service planning and decision making. The congregate model will focus on fostering community and bringing relevant services and supports to the tenant. The congregate model works from the principle that housing is a human right and is the foundation for stability in all other aspects of tenants' lives. Discharge based on negative outcomes is very unlikely

(e.g., extreme levels of violence). If a tenant is incarcerated for an extended period of time, they will be considered 'inactive' rather than discharged. If the support team decides that discharge is an appropriate course of action, a documented process will be followed. There is no time-limit imposed on tenancy.

Service intensity/capacity: In the first six months of the program, the Case Manager (or related staff) will make frequent visits to engage with tenants and build trust, and to ensure that the necessary community links are being established or maintained at the tenant's pace. The Case Manager will work with tenants to modify service plans (if applicable) on an ongoing basis as their needs change. All changes to service plans will be developed jointly by the Case Manager along with the tenant.

Staffing: Basic business management of the building(s) (i.e., housekeeping, property management, meal provision, etc.) will be funded through the operational budget provided by BC Housing or another partner organization. A minimum two staff will be present in the building 24-hours per day, 7 days per week. Additional staff may be required on the evening shift if significant crisis intervention and supports are needed. Clinical care will be provided by a team of primary care practitioners (nurse or family physician), psychiatry, counseling, case manager, a physician who can administer methadone, concurrent disorders specialist, and psychosocial rehabilitation (community integration and programming, etc.). Staff will be recruited, trained and supported within the organizational culture of the PHS.

Program organization & communication: Programming will be based on the philosophies and values of the PHS: helping marginalized individuals stabilize their lives by understanding and accepting the tenant's reality and through community development, ownership and integration.

Assessment & treatment planning: Significant issues in many domains of daily living will need to be addressed via on- and off-site supports. If appropriate, tenants will have an individual service plan that addresses risks, needs and strengths in order to help them maintain housing and gain independence. Community participation and reintegration will form the backbone of individual service plans. The plans will be created and modified in full collaboration with tenants.

Services: The Congregate housing condition will be developed to include services that are standardized and comparable in their range, intensity and likely effectiveness to those described in the ACT condition, above. Many clients will require medications for physical and/or mental health conditions; medications can be administered via an on-site program or an external delivery program or nearby administration site. Some clients may use IV substances; needles should be available on-site, whether via a formal exchange or a more individualized approach. Some tenants will need a meal program. An off-site meal program, available nearby, will be the most cost-effective option.

Many clients will need their finances administered. This is best provided via an external service but can be provided on-site. A money management plan may also be developed, including how and when benefits are received, and a budget for rent and other necessities. Staff will work with the tenant and the financial lead at PHS to ensure that all necessary payments are made on time to the client or any other payee necessary. A significant number of tenants will pursue education, employment and volunteer activities. These services will be provided externally, although on-site supports will encourage clients and help them problem-solve participation issues. Tenants will be encouraged to

participate in social activities in their communities that relate to their own experiences and interests. Staff will ensure that tenants are aware of peer supports in their communities, including groups and clubs that focus on clients' personal interests. Services will be embedded in a continuum that allows priority access for tenants to enter "next step" housing, if they wish.

For each of the interventions described above (i.e., ACT, ICM, Congregate), accurate and timely record keeping (based on an electronic health record system) is critical for all work and communications with or about a client. Record keeping should be completed within 5-7 business days of the service/meeting. Accountability to clients, landlords and the community is achieved via ensuring follow-through on roles, responsibilities and expectations and being accessible. The service provider lead organization will be responsible for the delivery of their respective intervention.

4.4.5 Treatment as Usual (TAU)

Given recent innovations in housing and supports in Vancouver, many opportunities for housing and supports will be available to individuals in the TAU condition. The Vancouver Coastal Health Authority and several Provincial Ministries have initiated innovative programs designed to improve outcomes for people with mental disorders, housing, and other diverse challenges in the DTES (e.g., Community Court, the Burnaby Centre for Mental Health & Addiction). The City of Vancouver and BC Housing have purchased eighteen SROs, and additional supported housing is being developed through additional partnerships. The Ministry of Employment & Income Assistance was recently reconstituted as the Ministry of Housing & Social Development, with responsibility to integrate housing and other publicly funded services. Monitoring the outcomes associated with this group is important for comparison to those in the intervention groups as well as to assess fluctuations in the course of homelessness.

4.5 Follow-up and Retention Procedures

Careful tracking will be critical to ensure a high response rate and to minimize attrition bias, particularly for the TAU group. Steps to retain participants will include regular contacts with all participants at 6-month intervals over the course of the 2 year follow-up and regularly updating contact information gathered from administrative data, participating service providers and public records. The TAU group will be encouraged to visit the field office on a monthly basis to check in with staff and to update their contact information. In addition to the remuneration they receive for participating in the five interviews, participants in this group will receive a $10 honorarium for each month that they check in. Food will also be available to further encourage field office visits. For those participants who cannot be located in-person or reached by phone, outreach workers in the community will inquire about their whereabouts. Locating these participants will require the full-time efforts of trained field workers (e.g., peer researchers) who are well-embedded in the community and are familiar with following homeless individuals over a period of time.

4.6 Outcome Measures and Timelines

Data collection will occur in three main areas, which are detailed below: (1) Physical and mental health (including substance use); (2) Psychosocial determinants of health and consequences of change; and (3) Cross-sector administrative data and cost outcomes. Unless otherwise indicated, data collection will occur at baseline and every six months for two years. A number of instruments are suggested in the following sections but will not be finalized until after discussion with the National Team. The focus will be on instruments that are used internationally and have sound psychometric properties. Please see Addendum B for a copy of all proposed instruments.

4.6.1 Physical and Mental Health

Mental health symptoms and severity (including substance use and all Axis I and II diagnoses) will be assessed using the Symptom Checklist (SCL-90) and the MINI or the Psychiatric Diagnostic Screening Questionnaire (PDSQ). Other instruments suggested for the assessment of substance dependence/abuse include the Global Appraisal of Individual Needs (GAINS) – Substance Problem Scale, the Diagnostic Interview Schedule (DIS) and the Opiate Treatment Index. Adverse events, stress, and trauma symptoms are additional variables of interest and could be measured using the START Outcome Scale[3], Childhood Trauma Questionnaire and/or Adverse Childhood Events Scale, and the Trauma Symptom Checklist-40.

Given the known high mortality and the dearth of data regarding physical health conditions among people who are homeless, 20% of participants (a random sample of 20 per group, for a total of 100) will be accompanied by peer researchers to a research office at St. Paul's Hospital where physical assessments will be coordinated. If participants are in treatment or have received a recent physical examination, we will ask the participant for permission to access relevant details from their health record. The specific assessments and timelines proposed for physical health examinations are summarized in Table 1. If a participant in any treatment group is identified as having a medical condition that is in need of treatment, appropriate referrals will be made. Participants will receive an honorarium of $50 for each physical examination.

Table 1. Proposed Physical Health Assessments and Timelines

Physical Health Assessments	T-1 (Inclusion)	T0 (Intake, Assignment)	T1 (+6)	T2 (+12)	T3 (+18)	T4 (+24)
Opiate Treatment Index		X	X	X	X	X
Chronic Health Scale		X	X	X	X	X
Physical exam [subsample] including electro-cardiogram, X-Ray, MRT, derma exam, basic blood work, infection screen (HIV, HCV, STD), basic urine toxicology, TB skin test		X				X

4.6.2 Psychosocial Determinants and Consequences of Change

Both qualitative and quantitative data will be collected through three core methods: semi-structured interviews, surveys/questionnaires and focus groups. The initial data will be largely descriptive. Over time, it will be possible to collect longitudinal data from health professionals, service and housing providers and clients. Whenever possible, data collection procedures will be triangulated to ensure accuracy and validity (e.g., base rates). All interviewers will be trained to administer the survey instruments in a consistent and standardized manner.

4.6.2.1 Surveys and Questionnaires

The survey instruments consist of previously validated measures recommended by the National Project Team. We have also proposed a number of additional relevant measures. Copies of instruments are presented in Addendum B. Please see Appendix 5.0 for a summary of the timing of administration.

Housing status over the preceding 6 months will be determined at baseline and at the time of each follow-up interview using the Housing Timeline Follow-Back Calendar (HTFBC). The primary outcome variable will be housing status at the time of the 2 year follow-up interview, defined as being either: (1) Homeless, or (2) Stable Housing (living for a continuous period of at least 90 days in a house,

[3] The SOS is based on the core of the modified Overt Aggression Scale (see Addendum for a copy of this instrument).

apartment, SRO hotel, rooming house, or any other place of one's own for which one pays rent; Ontario MCFS, 2004). Other outcome variables to be derived from the HTFBC will include: (1) housing status at each follow-up period; (2) number of housing transitions over the previous 6 months; (3) number of nights spent homeless over the previous 6 months. The Housing Quality Score will be used to determine self-reported quality of current living conditions in terms of comfort, safety, spaciousness, privacy, friendliness and overall quality.

Demographic characteristics will be collected using subsections of the Community Mental Health Evaluation Initiative (CMEI). Quality of life will be assessed using two instruments: (1) The Quality of Life for Homeless & Hard to House Individuals (QOLHHI) Instrument (Hubley & Palepu, in preparation); and (2) the EuroQol (EQ5D), a standardized health-related quality of life instrument that generates a composite score reflecting the preference value associated with a given health state, and a global rating of current health using a visual analog scale. General functioning will be assessed using the Multnomah Community Ability Scale (MCAS). Resiliency will be assessed using the 3-item Sense of Coherence Scale. Vocational outcomes will be assessed using a timeline follow-back interview from the Montreal IPS study (indicated by MHCC), which examines work status and various details of work (e.g., setting, timing, hours, compensation, etc.).

The Sarason Social Questionnaire (SSQ-Short form) will be used to measure perceived number of social supports and satisfaction with available social supports. Family history and critical life events will be assessed using items developed by Roy et al. for the Montreal Street Youth Cohort Study. The Objective Social Outcomes Index (SIX) will be used to assess social outcomes related to mental illness.

4.6.2.2 Semi-Structured Interviews

We propose to interview individual participants and, where possible, services providers, and health professionals in relation to our hypotheses. Interviews will be in-person or by telephone. A draft interview protocol will be developed in collaboration with the local/national team and will be pilot tested with clients to ensure clarity, appropriate length, inter-rater reliability and lack of redundancy. Experienced graduate students and/or service providers or peer interviewers will conduct the interviews. This component of the data collection should take about 90 minutes to complete.

Personal Stories: Following the baseline quantitative interviews, study participants will be invited to participate in a second set of interviews that will focus on their life story. Approximately 10 participants from each study "arm" will be randomly selected to participate in semi-structured interviews that will address personal experiences with homelessness and mental illness. An equal distribution of men and women will be attempted (20 from the moderate needs group and 30 from the high needs group).

Two personal life story interviews will be conducted. The first interview will take place approximately 30 days after the participant entered the study and will focus on particular events, memories or episodes in their lives (one high point, one low point, one turning point) that occurred prior to and in the early stages of their participation in the study. The second interview will take place 18 months later and will focus on specific experiences related to their housing, support and clinical services. For participants who have been randomized to one of the intervention groups, questions will focus on their experiences with service providers and will include seeking permission to also interview their service providers. For participants who have been randomized to one of the TAU groups, a parallel set of questions will focus on their experiences with community service providers.

Interviews will be conducted by graduate students or service providers paired with a peer interviewer. During the interviews, respondents will be encouraged to address each topic in their own way and using their own words. This approach is essential for obtaining valid data on potentially sensitive issues by facilitating rapport between interviewer and participant. An open-ended approach allows participants to introduce substantive topics not considered by researchers (Berg 1995). Interviews will last approximately two hours and, with permission, will be recorded for full transcription and analysis. If participants refuse permission to record, researchers will take detailed notes during the interview. An honorarium of $50 will be provided for participation.

Qualitative interviews and focus groups will be conducted to illuminate issues regarding individuals' experience of homelessness and service provision. Interviews will be conducted with a subset of randomly selected participants, staff, and other key stakeholders as outlined in the RFP.

4.6.2.3 Focus Groups

It is important to examine group perspectives on homelessness. Focus groups will be used with subsets of participants, service providers and key stakeholders to gain a better understanding of homelessness, mental illness and service provision/utilization. We propose an annual focus group with clients randomly selected from each housing/treatment condition. Each group will begin with an introduction followed by a discussion about participants' behavioural intentions for dealing with psychosocial issues. A facilitator and a recorder (trained collaborators or graduate students) will lead each group. Key points will be recorded on flip charts and audio-taped for in-depth analysis. The facilitator and recorder will meet before and after each session to ensure quality and consistency.

Planning & Proposal Development: Data will be gathered in the following areas via focus groups, key informant interviews, and review of program planning documents: (a) Planning processes; (b) Principles and values guiding the planning process; (c) Planning actions, and (d) Stakeholder relationships. This data will be gathered by graduate research assistants during the recruitment and baseline data gathering phase (Sept/09 – March/10).

Implementation Phase: Data will be gathered from key stakeholders in the housing and clinical components of the project in the following areas: (a) Implementation processes; (b) Principles and values guiding the implementation process; (c) Implementation actions; and (d) Stakeholder relationships. Data collection will include focus groups, key informant interviews, and review of program planning documents after the project has been running for one year (September 2010).

4.6.3 Cost Outcome Analyses & Administrative Data

All study participants will be asked to provide consent for the research team to utilize their personal identifiers (i.e., name, date of birth, personal health number) to obtain administrative data reflecting their health care utilization (hospital, physician services, prescriptions), social services utilization (income assistance, disability and unemployment benefits) and criminal justice encounters (arrests and Provincial sentences). The research team at CARMHA has extensive experience collaborating with government agencies in the linkage and analysis of these data. The costs associated with service events have been established through previous studies by CARMHA and the Provincial Government, and will be the basis for econometric analyses in the current study.

Data will be sought retrospectively to 1996, enriching our understanding of the trajectories of service utilization preceding study participation. These data will be refreshed and updated each year through

the active study period (2009-2013), and then bi-annually until 2019. Funds to support research following the study period will be sought separately.

4.6.4 Additional Outcomes of Interest

The core data collection will be leveraged for other outcomes of interest. Participating service providers and health professionals will be asked to complete the Health Professionals' Attitudes to Homeless Inventory (HPATHI) which assesses comfort/attitudes, interest, and confidence of health professionals in work with homeless and at risk persons.

Employment and income assistance trajectories will also be examined over time, with particular attention paid to the experiences of women. These will be important outcome measures given their contribution to individual self-sufficiency and housing stability.

4.7 Good Clinical Practice

As an RTC, by design, we are obliged to comply with the Guidelines for Good Clinical Practice (GCP; 1996), an international ethical and scientific quality standard for designing, conducting, recording and reporting trials that involve human subjects. Components of GCP are addressed at various points throughout this application; however, a thorough review of GCP, including Standard Operating Procedures, training, reporting, external audits, will need to be developed for all three intervention conditions as well as the TAU group outside the scope of this application.

4.8 Data Integrity and Management

All data will be retained in established, secure data facilities created at CARMHA at the downtown campus of Simon Fraser University. Data collection infrastructure will be provided by a professional information systems vendor and will include equipment for field data collection, secured mechanisms for transmission and processes for quality control. Protocols for data security and integrity are maintained by CARMHA and would be extended to include the data gathered from this study.

- Processes for the involvement of consumers as co-investigators and interviewers
- Partnerships with multiple Provincial Ministries, health agencies, and non-profits in order to gather, maintain, and analyze primary data and linked administrative data
- On site facilities for secure data input, verification, archiving, back-up, analysis
- Expertise on site to design and direct chain of custody for all data, sharing with relevant investigators in Vancouver and the National team

We will also develop additional resources for key operational tasks, including data collection and entry (graduate research assistants); data verification and management (Data Analyst/Manager); analytic consultant(s) specific to distinct methodological areas, including path analysis and econometrics (e.g., health economist); and additional computational resources, as needed.

4.9 General Approach to Data Analysis

Quantitative Data Structure

The data structure for the quantitative analyses is outlined in Table 2 below (which presents the general structure of this data file for a single participant [case 01] who is assumed to have been interviewed in each of the five waves of data collection). The first row (shaded) shows the structure of the baseline study used for cross-sectional analyses. The five rows taken together represent the repeated data over the course of the study period. All analyses will be based on an intent to treat.

Table 2: Structure of the RCT Longitudinal Quantitative Data

Case ID	Time	Stable Covariates	Time-Varying Covariates	MH/SA Status	Homelessness Status	Service Utilization
01	Baseline	**X**	$\mathbf{Y_1}$	$\mathbf{Z_1}$	H_1	$\mathbf{S_1}$
01	6 mos	**X**	$\mathbf{Y_2}$	$\mathbf{Z_2}$	H_2	$\mathbf{S_2}$
01	12 mos.	**X**	$\mathbf{Y_3}$	$\mathbf{Z_3}$	H_3	$\mathbf{S_3}$
01	18 mos.	**X**	$\mathbf{Y_4}$	$\mathbf{Z_4}$	H_4	$\mathbf{S_4}$
01	24 mos.	**X**	$\mathbf{Y_5}$	$\mathbf{Z_5}$	H_5	$\mathbf{S_5}$

Definitions:
X = A vector of variables and/or indicators representing individual characteristics whose values do not change over time. Examples include gender, age at entry, race/ethnicity and group intervention.
$\mathbf{Y_i}$ = A vector of variables and indicators whose values at baseline may need to be repeatedly updated. Examples are psycho-social indicators such as family structure, income, indicators of social capital (family, marital status and social network indicators), human capital (schooling and employment status), indicators of resiliency (sense of coherence) and other events (e.g., a serious illness or injury) that occurred in the 6months prior to the ith interview.
$\mathbf{Z_i}$ = A vector of indicators of alcohol, drug and mental health status as of the ith interview. The time frame for these measures extends from "past week" or "last 30 days" to "last 6 months".
$\mathbf{H_i}$ = A vector of indicators of housing circumstances in the 6 months prior to the ith year interview. See the discussion of these measures under Aim 1 below.
$\mathbf{S_i}$ = A vector of indicators of services used during the 6 months prior to the ith interview. These include services connected to receipt of the interventions (e.g., substance abuse treatment or work programs). These data will consist of information from records as well as self-reports.

Quantitative Data Analysis

In its most basic form, each RCT can be viewed as a mixed-effects 2 by 5 model (or in the case of the high needs group, a 3 by 5 model) with a number of different outcome measures of interest based on specific hypotheses (see section 2.3 for the outcome measures) and a number of potential covariates. The hypotheses address comparisons of specific sub-groups within the overall model.

The general analysis plan proposes several broad strategies of data analysis that will build upon the data as it is collected over the study period. The approach to analysis will begin with a baseline examination of the distribution of key factors across each sub-group. This univariate comparison of the distributions of key individual characteristics (e.g., demographic characteristics, substance abuse and mental health indicators) across each RCT sub-group will provide for an assessment of the effectiveness of the randomization process as well as identification of potential confounding factors that need to be accounted for in further analyses. Preliminary steps for all analyses will also involve assessing bivariate cross-tabulations and correlation matrices for relationships among variables. All variables will be checked with regard to their distribution (e.g., for normality and departures from normality) using frequency distributions, scatter plots and histograms. Statistics such as means, standard deviations, ranges, modes, medians and estimates of skewness and kurtosis will be computed.

We propose to assess our primary and secondary hypotheses using two approaches. The first (and simplest) is to perform cross-sectional analyses at each of the five measurement points (baseline, 6 months, etc.) comparing the 5 distinct intervention groups on the measure of interest at each point in time. Depending on the measure, this can be done using a non-parametric comparison for the categorical measures such as physical health status or parametric comparison (with confidence intervals) for the continuous measures (such as number of days housed). Plotted over time, this approach can provide a straight- forward and visual assessment of the relationship between the various interventions and the outcomes of interest. This approach, however, does not take into account the role of time or the variation within-groups over time specified in the hypotheses. Thus, we propose to conduct a number of longitudinal repeated measures analyses with the choice of the exact model depending on the intent of the individual hypotheses. We will begin with Generalized

Estimating Equations (GEE), adding in the specific interaction terms of interest as specified in the various hypotheses. This approach is based on specific assumptions around the pattern of missing data and will be adjusted as need be. An alternative approach is in the assessment of individual trajectories and draws from random effects models. Random effects models allow for a measure of variability in individual trajectories around the average group trajectory and are especially useful when it is reasonable to think of individual trajectories as a sample from the population of homelessness trajectories. All models will be implemented utilizing software applications which have the capability of modeling complex error structures at both the across individual (e.g., each person has their own trajectory of homelessness) as well as the within-individual (e.g., auto-correlated error structures of individuals' responses within their across time homelessness trajectory) levels.

Analytic Limitations: *Missing Data.* A concern throughout the analyses is the effect of missing data due to item non-response as well as panel attrition. In the case of missing data due to item non-response among persons who participate in a cycle of data collection, we plan to implement imputation methods, particularly for critical variables utilized in the cross-sectional presentation of the results. In general, we will use multiple imputation methods (Rubin, 1987) involving MCMC simulations. Available methods (Rubin, 1987) will be used to combine analysis results from multiple imputations that incorporate the uncertainty due to item non-response in the estimates of parameter variances. Models based on GEE (or related) approaches do not require the estimation of missing data.

Panel attrition. This is often more influential than item non-response on the comparison of groups. We will begin with a comprehensive investigation of baseline characteristics associated with patterns of participation will be examined by study follow-up wave to help identify the characteristics associated with trends in participation over time. Variables found to be significantly associated with attrition will be included as covariates in the models.

Power considerations. In light of the well-defined criteria established by the MHCC surrounding the sample size of each intervention arm as well as the understanding that the MHCC criteria takes account of panel attrition, no power calculations were conducted within the context of this application.

*Administrative Data***.** The administrative data will be linked and incorporated with the quantitative data based on the appropriate vector of variables (see Table 2 above). Quantitative data analyses will be conducted drawing from these data in the same manner as that of data collected in the study.

Qualitative Data. We will begin constructing the qualitative database by entering and coding field notes, and transcribing the audio-taped interviews using NUD.IST and/or NVIVO software. One of the PIs (JF) has extensive experience in qualitative data analyses and has successfully used this software in prior studies. Simultaneous and iterative conduct of data collection and coding is central to qualitative research as it allows investigators to take advantage of insights that emerge during the course of fieldwork (Strauss, 1987). As data accumulates, the coding scheme will change to reflect the generation of new insights and the accumulation of increasingly detailed information. Thus, the outline presented above is an initial coding scheme that will change and expand over the course of the project.

Once we have identified and coded field note and interview data, the next step in analysis will be to distinguish repeated patterns from idiosyncratic individual statements and actions. This will be done by identifying which codes appear only infrequently in our database and which recur in a wide variety of interview transcripts and field notes. To enhance reliability, multiple analysts will examine the content of coded statements to ensure their accuracy and coding discrepancies will be identified

and addressed. Conducting analysis simultaneously with ongoing fieldwork will allow us to follow up on important statements, observations, and behaviors in later interviews and fieldwork (Glaser & Strauss, 1967). As observations and interviews progress, we will compose analytic memos that summarize the in-progress findings in the qualitative database, and help to identify areas for ongoing fieldwork. As the database grows, we will begin to identify similarities and differences across service settings and different groups of participants in the study.

5.0 Ethical Considerations

This project will be conducted in accordance with the Research Ethics Boards of SFU, UBC and the Tri-Council Policy Statement on Research Involving Human Subjects. The research team at CARMHA has extensive experience managing large databases of sensitive information. No identifying personal information about the study participants will be kept with any of the interview or administrative data. All electronic and paper records will be maintained using existing protocols and facilities at SFU Harbour Centre. Dedicated server rooms, work stations, and filing systems are all maintained in a secure environment that is audited for security and compliance with best practice. Records linking participants' study identification number and personal data will be kept under lock by the Research Coordinator at CARMHA, in a location separate from where questionnaires will be stored. Identifiers will be removed from all data as soon as possible. Furthermore, all research personnel who have access to raw data will be bound by confidentiality agreements.

5.1 Quantitative Data

Risks to subjects. ***Human subjects involvement.*** Baseline interviews will be conducted with 500 participants with four follow-up interviews over a period of 2 years. All participants will be of legal adult status. Potential risks associated with the interviews are embarrassment and discomfort from discussing matters such as personal mental health problems or drug use. However, past research with similar surveys indicates that such risk is minimal. Further, these potential risks can be minimized by training interviewers carefully and by assuring subjects that interviews will be conducted in private. Another potential risk involves updating contact information at each follow-up interview with respondents who had nearly been lost to follow-up. This information is given voluntarily, and respondents will be familiarized with this process.

Within the context of this study, it is expected that the mental health problems and symptoms of both the moderate and high needs groups will diminish over time. Given the target population and the chronic nature of mental illness, it is likely that some individuals will, over time, move between the high and moderate needs group. However, there is a possibility that some individuals in the moderate need group could experience a significant escalation in their symptoms. In this event, the individual's case will be brought forward to an as yet to be determined committee of researchers and service providers, including a physician, in order to evaluate whether or not the individual or others are in significant danger. In the event that the committee determines the individual is a risk to self or others, the individual will be referred for more intensive treatment.

Physical health examinations (PHE) will be completed with a subset (20%) of study participants at baseline and at 24 months following baseline. PHEs will be carried out by qualified physicians who have expertise providing care to homeless and seriously ill urban patients. Participants will have the option of refusing or discontinuing PHEs at any point. The PHE are anticipated to reveal untreated or undertreated health conditions, which may require immediate or urgent treatment. Good clinical practice will govern the follow up and treatment of medical conditions identified in PHEs.

Adequacy of Protection Against Risks. *Informed Consent*. Prior to the baseline interview, all respondents will be evaluated for competency with regard to their ability to understand informed consent. Once competency has been established, signed informed consent will be obtained from all respondents. A lengthy consent form will be read to the respondent authorizing consent to be interviewed, to be randomized to a housing and services group, to have administrative records accessed by the study team, to participate in the qualitative interviews, and to be re-contacted for the follow-up or longitudinal tracing through personal contact information and through public records. As part of the informed consent process, individuals will be informed that: (a) participation is voluntary, and that there are no consequences for refusal to participate; (b) they could be randomized to a condition of 'usual care'; (c) participation will involve answering questions about their background, health problems, housing circumstances, and their experiences with services; (d) even after consenting to participate, participants can interrupt the interview at any point or refuse any treatment, services or to answer any question; (e) all responses and results will be entirely confidential, and no identifying information will appear in any material associated with the study; and (f) no information about respondents as individuals will be shared with parties outside the research team. At each subsequent follow-up interview, verbal consent will be obtained after providing study participants with a description of the general content of the interview, notifying them that all information will remain completely confidential, and that participating in the study will be completely voluntary. After the interview, information relevant to tracing and the next follow-up will obtained. Respondents will receive a $40 honorarium for their participation in each quantitative interview.

5.2 Qualitative Data.

Human Subject Involvement. The second component of this study includes in-depth interviews (one approximately 30 days following baseline study enrollment and the other approximately 18 months later) with 50 participants from the RCT. Respondents will be encouraged to address topics of interest using their own words. The baseline interview protocols, yet to be developed, will have an introductory discussion of the respondent's background and current situation. After establishing rapport, interviewers will turn to potentially more-sensitive topics, such as attitudes towards homelessness, substance abuse and interactions with service providers. Participants will receive an honorarium of $50 for their participation.

Potential risks. This study component involves minimal risks to participants. Again, potential risks associated with the interview are embarrassment and discomfort in providing information about matters such as homelessness, mental health and drug use. However, the research team's past experience with similar populations indicates that such risk is minimal. Furthermore, these potential risks will be minimized through interviewer training and by assuring that the interviews will be conducted in private. Since we do not require respondents' names for this component of the study, we will seek a waiver of written informed consent and develop an oral informed-consent procedure.

Adequacy of protection against risks. *Informed consent and confidentiality*. All required components of informed consent will be included and reviewed with participants. To further minimize risks, we will maintain strict respondent confidentiality by conducting interviews in private locations, by employing an ID system to assure that respondent names are never associated with transcripts, by storing interview tapes and transcripts in secured locations and password-protecting computer files. Only select research staff will ever have access to identifying information.

5.3 Administrative Data.

Risk to Subjects: *Human Subject Involvement*. All study participants will be asked to provide consent for the researchers to utilize their personal identifiers (i.e., name, date of birth, personal health number) to obtain administrative data reflecting their health care utilization (hospital, physician services, prescriptions), social services utilization (income assistance, disability and unemployment benefits) and criminal justice encounters (arrests and Provincial sentences). Data will be sought retrospectively to 1996 and will be refreshed and updated each year through the active study period (2009-2013), and then bi-annually until 2019.

Potential risks. This study component involves minimal potential risks to participants. However, a potential risk associated with collection of the administrative data surrounds embarrassment and discomfort in the event that the individual's identity is compromised. In order to minimize this potential risk, the electronic dataset used for the analyses proposed here will include *no personal identifying information.* Results will be reported in ways that do not reveal respondent identities.

Adequacy of protection against risks. *Informed consent*. During the consent procedures we will emphasize that: a) participation is completely voluntary; b) even after consenting to participate, they may refuse access to their administrative data; c) and no personal identifying information will appear in any materials associated with the study. No identifying personal information about the study participants will be kept with any of the administrative data. Records linking participant's study identification number and administrative data will be kept under lock by the Project Manager at CARMHA. All personal identifiers will be removed from the administrative data as soon as possible.

5.4 Procedures for REB approval

Upon notice of grant award, an application for human subjects approval will be submitted to the Research Ethics Boards at SFU and UBC. The applications will be submitted simultaneously and will be identical with the exception of institutional requirements that vary. The application for ethics approval will include both the RCT studies as well as a request to utilize and publish the results drawn from the key informant interviews with persons with lived experience that were conducted under contract with the MHCC. Contact with human subjects will not proceed until REB approval has been obtained from both SFU and UBC.

6.0 Sustainability

We are committed to ensuring that all participants are able to maintain their housing and receive the supports required beyond this project. We have had initial discussions with BC Housing regarding the sustainability of housing subsidies for those in scattered-site units as well as possible transitional options for people housed in the congregate setting. It is possible that we will not be able to secure sufficient ongoing housing subsidies for all participants and transition to alternative housing and supports may be necessary. We will work with individuals and link to the BC Housing Coordinated Access to Housing team in Vancouver to ensure successful transitions as needed. It is anticipated that a significant number of the new housing units currently under development will be available both before and after the end of the project and we will explore these options with BC Housing as they become available. We will continue to forge a partnership with the Streetohome Foundation and link to its work on housing and service reform locally. We have also held initial conversations with the BC Ministry of Health to explore ways to ensure sustainability of funding for the services required for participants post-project, particularly with respect to the ACT intervention. Through engagement of our advisory and oversight committees as well as through meetings with provincial and local decision makers, academics and service providers, and people with lived experience, we will create and sustain

momentum for the project. The local oversight committee, composed of senior decision-makers will also provide a mechanism to leverage funds both during and post-project. It will be critical to actively engage additional key stakeholders locally in order to successfully build a strong local environment for sustainability. In this regard, Vancouver Coastal Health Authority and Providence Health Care – which operates the hospital that provides the majority of emergency and acute hospital-based services to people who experience homelessness and mental illness – are key partners with whom we will need to build strong and formal partnerships both at a governance and operational level. Both organizations have been well represented throughout the Consortium development process. Now that we have identified service leads, it will be critical in the next month to work with both Vancouver Coastal Health Authority and Providence Health Care to clarify their respective roles in supporting implementation of all three of our identified interventions. Moving forward, we will actively explore evidence-based models such as networks/communities of practice to facilitate service integration and knowledge exchange within and between relevant partners and stakeholders.

7.0 Integrated Knowledge Translation Strategy

Effective knowledge translation (KT) is a central goal of this study. We are committed to engaging researchers, research users and people affected by research at all phases of the project including developing questions, defining research methods and interpreting and disseminating results. Our strategy will include knowledge dissemination and dialogue about the research process, findings and policy implications, aimed at a wide range of audiences. The Vancouver site is committed to participating in any cross-site knowledge translation activities as required.

Our team of investigators and collaborators has strong connections to a wide range of research, policy, government, institutional, service-provider and advocacy networks, operating at multiple levels. Our community-based collaborators are leaders of best practices within their field and strong advocates of health equity and change. Their experience in public education, advocacy and their well established community coalitions, media contacts, and policy networks will enable them to be a vital conduit for translating research findings into policy and program implications. KT will also be achieved through activities such as managing partnerships, implementing training and professional development sessions (e.g., on-line training, communities of practice), a frequently updated website, sharing of partnership best practice, providing partnership expertise to all stakeholders involved, and developing and implementing a framework and tools for collecting input from stakeholders. People with direct experience will be centrally involved in all KT activities both as members of the advisory committee and as integral parts of all KT initiatives (i.e., symposia, public stakeholder engagement and ongoing ad hoc round tables regarding ethical considerations, service process and sustainability strategies).

Initial conversations have taken place with the Provincial Health Services Authority who has specific responsibilities in KT provincially. It is envisioned that the project will be able to partner with PHSA effectively to ensure strong and meaningful KT throughout the project. CARMHA and the local site coordinator are involved in the ACT Provincial Advisory Committee and will work with the committee to share learning, build communities of practice and focus on ACT implementation and evaluation.

A central focus of our strategy will be to synthesize research findings into clear and meaningful messages that are tailored to specific stakeholder audiences and their concerns. Our findings will be disseminated through written materials aimed at a variety of audiences including peer-reviewed journals, plain language reports, and media releases. Our team will also engage in a range of

interactive strategies to engage key stakeholders, including presenting our findings at academic, community and policy conferences and workshops; meeting with key decision makers; engaging in media work; and organizing a series of research dialogues in Vancouver. A comprehensive KT strategy will be developed in collaboration with the National Knowledge Broker during the implementation phase of the project.

7.1 Media and Communications

A detailed communication plan will be developed that includes the general public, public affairs/government, internal relations, and media relations. For SFU-affiliated staff, local media and communications will be managed through the President's Office and SFU Media Relations, collaborating in full with the National Communications Liaison. Media relations will be focused around proactive issues that are identified at the Project Team level, Advisory or Oversight Committees. A formal protocol detailing key spokespersons will be developed and monitored. Key messages will be defined and confirmed collectively whenever possible in order to ensure consistency and quality of messaging. A comprehensive communications strategy will be developed in concert with the National communications person during the course of the implementation phase.

8.0 Project Structure and Governance

8.1 Committees/Teams

The following committees/teams will be necessary for this project:

Committee	*Purpose of Committee*	*Membership*	*Timeframe / Frequency*
Project Team	• Provides project direction & retains operational responsibility locally<br>• Develops and implements a project management structure<br>• Facilitates the development of memos of understanding<br>• Develops and uses an issue-resolution mechanism<br>• Accountable for day-to-day management & execution of the project.	• PI<br>• Co PIs<br>• Site Coordinator (Chair)<br>• Research Coordinator<br>• ACT Lead<br>• ICM Lead<br>• Housing Lead<br>• Congregate Lead	Bimonthly (more or less frequently if needed)
Research Team	• Provides research guidance and expertise	• PI & Co-PIs<br>• Co-investigators<br>• Site Coordinator<br>• Research Coordinator	Bimonthly (more or less frequently if needed)
Sub-Research Teams (Physical Health; Psychosocial; Administrative Data)	• Design, conduct and analyze research in respective areas	Individual research teams (Project Manager/ Coordinator; research assistants, data analysts, etc.)	Weekly
Advisory Committee	• Provides strategic advice and	• People with Direct Experience	Quarterly

Committee	*Purpose of Committee*	*Membership*	*Timeframe / Frequency*
	feedback to Project Team<br>• Provide as needed help with operations<br>• Information feedback loop for Project Team and member agencies	• Government<br>• Health Authority<br>• Service Providers<br>• Community Agencies	
Oversight Committee	• Ensure high-level awareness and buy-in<br>• Discuss sustainability issues (leveraging funds), systems-level changes, etc.	CEOs of health and housing organizations	Twice annually
Housing Portfolio Lead (MPA)	• Secure 200 scattered-site units<br>• Administer rental subsidies<br>• Build and manage relationships with landlords<br>• Property management	• David MacIntyre<br>• Sue Baker<br>• Housing portfolio development staff	Weekly
ACT Team (Rain City Housing)	• Establish team<br>• Ensure service delivery,	• Greg Richmond<br>• ACT team staff	Daily
ICM Team (Coast Mental Health)	• Establish team<br>• Ensure service delivery	• Darrell Burnham<br>• Tracy Schonfeld<br>• ICM team staff	Weekly
Congregate + Supports Team (Portland Hotel Society)	• Establish team<br>• Ensure service delivery, etc.	• Liz Evans<br>• Congregate housing and supports staff	Weekly

8.2 Scientific Advisors

A number of senior academics have agreed to provide high-level scientific advice throughout the project including Dr. Alan Marlatt, University of Washington; Dr. Brian Rush, Centre for Addictions and Mental Health; Dr. Ernest Drucker, Montefiore Medical Center and Albert Einstein School of Medicine.

8.3 Project Management Capacity/Structure

CARMHA has demonstrated capacity to administer large, interagency research initiatives. Further, all consortium members have established records of multi-agency, multi-disciplinary research projects. Given the Consortium's decision to not select an overall service lead (see Section 2.2), key service coordination and service-research coordination responsibilities have been assigned to the local site coordinator. Additional staffing resources will be required by the site coordinator to ensure sufficient project management capacity and support.

8.4 Project Organization Chart

The following illustrates the reporting structure of the project.

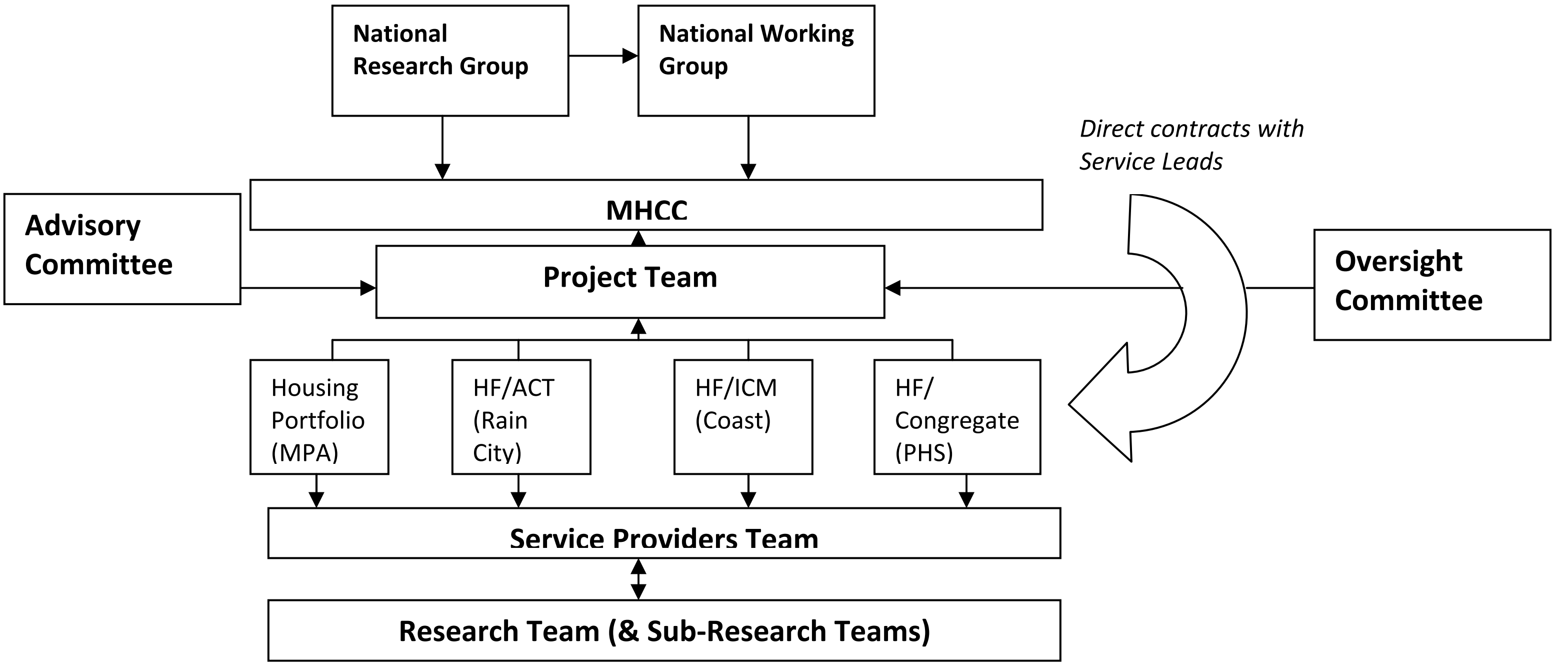

8.5 Roles and Responsibilities of Stakeholders and Project Team

The following descriptions define the general roles and responsibilities of the resources to support this project.

Role	*Responsibilities*
Principal Investigator	❑ Attend meetings of Project Team<br>❑ Ensuring the delivery of the final product and all interim deliverables as agreed in the RFP ;<br>❑ Liaising with National Team and other sites.
Co-Principal Investigators	❑ Attend meetings of Project Team<br>❑ Responsible for delivery of products within sub-project or component.<br>❑ Identifies resource requirements within sub-projects
Site Coordinator	❑ As part of the Project Team, plan and implement project activities<br>❑ Identifies and acquires resources<br>❑ Manages issues, decisions, changes, and problems to resolution.<br>❑ Communicates status and project information to sponsor, committees, and project stakeholders on a regular basis as agreed.
Research Coordinator	❑ As part of the Project Team, plan and implement research activities<br>❑ Manages issues, decisions, changes, and problems to resolution<br>❑ Ensures all project tasks and deliverables conform to quality management standards where they exist and are appropriate to the project
Co-Investigators	❑ Provide guidance and expertise on research-related issues<br>❑ Participate in Research Team meetings as needed
Service Leads (MPA, Rain City, Coast, PHS)	❑ Share seat and attendance at National Working Group<br>❑ Work with Site Coordinator to collaborate with other Service Leads and

Role	Responsibilities
	researchers<br>❑ Serve as a contact for their particular intervention<br>❑ Administer the budget for their intervention<br>❑ Administer housing subsidies (led by MPA)<br>❑ Develop and implement their intervention according to fidelity standards (or as defined nationally with local adaptations)<br>❑ Hire, support & supervise a services/supports team<br>❑ Ensure an enabling environment for researchers
Consortium Members (Health Service, Housing & Community-based Services)	❑ Provide guidance and expertise re: service delivery to the Service Leads and to the Research Team, as needed
Sub-research team leaders:<br>Denise Zabkewicz<br>TBD	❑ Completes the assigned tasks at the scheduled time as indicated in the project plan<br>❑ Manages issues, decisions, changes, and problems to resolution at the sub-project level<br>❑ Collects status from team and communicates to project manager on a regular basis as agreed<br>❑ Participates in management of project.

9.0 Workplan

Milestone	Estimated Completion Date
**Year 1** (Commencing April 1, 2009)	
Recruitment of key research personnel (Research Coordinator, Research Assistants, Peer Interviewers, Programmers/Data Analysts)	April – July 2009
Submit (and refine) Research Ethics Application (SFU and UBC)	April – August 2009
Develop Good Clinical Practice Guidelines (e.g., Standard Operating Procedures)	April – August 2009
Registration of Randomized Controlled Trial	April – August 2009
Secure recruitment sites and a community-based research office	April – August 2009
Service Planning and Implementation<br>- Infrastructure building and commitment<br>- Relationship building and communication (including team building and development of joint leadership)<br>- Continuous quality management<br>- Service delivery practices	April – Dec. 2009
Finalization of screening, intake, and outcome measures (including discussions with National Project Team),	April – July 2009
Purchase equipment and set up software	April – June 2009
Training of research staff in administration of measures (including qualitative interviews)	July – Aug. 2009
Recruitment of Participants<br>- Screening<br>- Intake and group assignment<br>- Physical examinations	Sept./09 – Mar/11

Milestone	Estimated Completion Date
Training and initial validation of interventions<br>- Local training<br>- Joint site training	April – Dec. 2009
Establishment of initial core of housing units and sites<br>- Scattered-site units (100 units)<br>- Congregate units (approx. 50 units)	April – Dec. 2009
Conduct baseline personal narrative interviews	Sept. 2009 – Sept. 2010
Gather data on planning process	April – Dec. 2009
Integration of ACT Team with Provincial ACTPAC	Sept./09 – May/10
Creation of Linked Administrative Database	January – April 2010
**Year 2** (Commencing April 1, 2010)	
Analyze qualitative data from client narrative interviews	Jan. – March 2010
Complete qualitative report on Planning & Proposal Development	April 2010
Complete acquisition of housing units and sites<br>- Scattered-site units (100 additional units)<br>- Congregate units (possible second building)	
Primary Data Collection #1: Completion of All Baseline Measures (Wave 1)	Oct. 2010 (based on 12 months to full sample)
Fidelity Verification ACT, ICM, Housing First	Sept. 2010
Retrospective Analysis of Linked Administrative Data	April – July 2010
First Annual *Housing First & Housing For All* Symposium: Envisioning Success	July – August 2010
Conduct Follow-up (18 month) Personal Narrative Interviewers	July – Nov. 2010
Gather and analyze qualitative data for Implementation Phase	July – Dec. 2010
**Year 3** (Commencing April 1, 2011)	
Primary Data Collection #2: Completion of Follow-up Data Collection (Wave 2)	April 2011
Completion of sample recruitment	April 2011
Complete data entry for all baseline measures	May 2011
Primary Data Collection #3: Completion of Follow-up Data Collection (Wave 3)	Oct. 2011
Second Annual *Housing First & Housing For All* Symposium: Lessons Learned	July – Aug. 2011
Sustainability Planning	(ongoing but intensified in end of Year 3 and through Year 4)
**Year 4** (Commencing April 1, 2012)	
Primary Data Collection #4: Completion of Follow-up Data Collection (Wave 4)	April 2012
Primary Data Collection #5: Completion of Follow-up Data Collection (Wave 5)	Oct. 2012
Third Annual *Housing First & Housing For All* Symposium: Preserving What Works	July – Aug. 2012
Analysis and Major Project Report	Jan. – April 2013

10.0 Budget (See Attached Spreadsheets)

11.0 References (See Appendix 6.0)
